# Microdevice for confinement of T-cells on functionalized bio-interfaces†

**DOI:** 10.1039/d5lc00248f

**Published:** 2025-04-22

**Authors:** Christoph Trenzinger, Caroline Kopittke, Barbora Kalousková, Nemanja Šikanić, Marina Bishara, Gerhard J. Schütz, Mario Brameshuber

**Affiliations:** a Institute of Applied Physics, TU Wien Wiedner Hauptstraße 8-10 1040 Vienna Austria ctrenzinger@hotmail.com brameshuber@iap.tuwien.ac.at

## Abstract

Mechanical stimuli are an integral part of the natural cellular microenvironment, influencing cell growth, differentiation, and survival, particularly in mechanically challenging environments like tumors. These stimuli are also crucial in the T-cell microenvironment, where they play a role in antigen recognition and pathogen detection. To study T-cell mechanobiology effectively, *in vitro* methods must replicate these mechanical stimuli induced by compression, tension or shear flow, in the presence of antigen-presenting cells (APCs). While custom-made microdevices and microfluidic chips have successfully observed bulk cell behavior under mechanical strain, no existing device fully replicated the T-cell mechanoenvironment comprehensively. In this study, we developed a microdevice that integrates the mechanoenvironmental aspects of an APC mimicry with compression under live-cell imaging conditions. This device allows for precise confinement of cells between two glass surfaces, which can be individually coated with functional bio-interfaces. The microdevice is reusable and enables presetting of confinement heights, manual seeding of cells and the assembly of components directly at the microscope. To validate our microdevice we confined primary mouse T-cells on different APC-mimicking supported lipid bilayers while monitoring their morphology and migratory behaviour over time. To study the effect of confinement on TCR signalling, we tracked intracellular calcium levels and quantified Erk1/2 phosphorylation by immunostaining. We observed that T-cell morphology and motility are affected by confinement but also by bilayer composition. Moreover our findings suggest that confinement, despite not interfering with T-cell activation, might increase TCR background signalling in resting T-cells. Importantly, our microdevice is not limited to T-cell research; it can also serve as a platform for studying mechanical stimulation in other cell types, cell aggregates like spheroids and organoids, or even tissue samples in the presence of various bio-interfaces.

## Abstract 2

Our microdevice integrates mechanoenvironmental aspects of cellular mimicry with compression under live-cell imaging conditions. It is reusable and allows for preset confinement heights, manual cell seeding, and on-microscope assembly of components.

## Graphical abstract

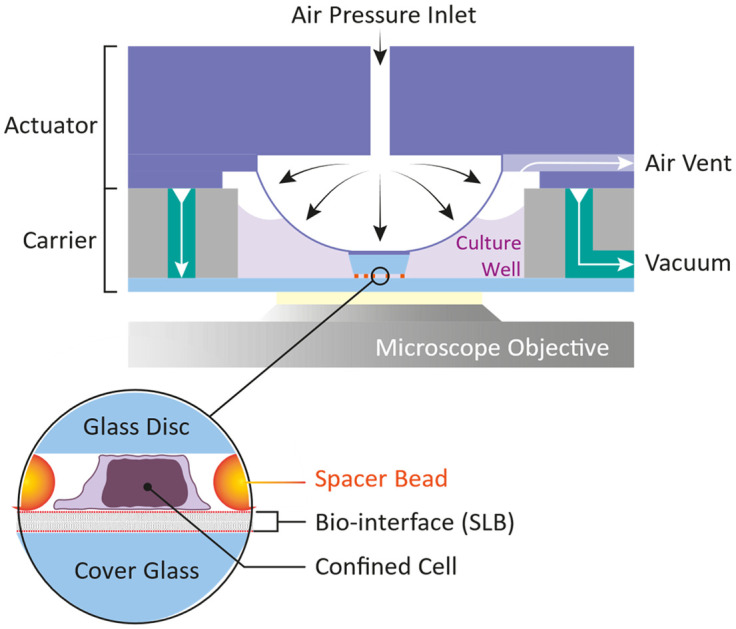

## Introduction

Mechanical stimuli are part of the cellular microenvironment. Mammalian cells are continuously exposed to shear stress, compression, and tension during migration and interaction with other cells and the extracellular matrix in body tissue, blood vessels, and the lymphatic system. Cells generally respond to mechanical cues through a process called mechanotransduction, which converts mechanical stimuli into biochemical signals. This process influences genome organization, gene expression and transcription.^1^ Multiple mechanotransduction pathways have been identified in immune cells, highlighting the role of the cellular microenvironment in regulating immune responses.^2^ This particularly applies to T-cells. As important players in the adaptive immune system, they patrol body tissues to search for pathogens. With the help of their membrane-associated T-cell receptor (TCR) complex, they discriminate self from foreign MHC-bound peptides (pMHCs) while scanning the surface of antigen-presenting cells (APCs). Notably, a T-cell can identify a single antigenic pMHC among thousands of endogenous peptides that are structurally similar.^3^ During transient TCR–pMHC binding events within the immunological synapse, tensile forces have been measured along the TCR–pMHC binding axes.^4,5^ Whether and how these forces contribute to the T-cells' outstanding sensitivity in antigen discrimination is still under debate.^6^ Recent studies suggest that the nucleus itself can act as a force sensor at the cellular level.^7,8^ Leukocytes have been shown to discriminate pore size using their nucleus as a mechanical gauge,^9^ which helps them to determine their migration path of least resistance. Confinement-induced responses allow immune cells therefore to adapt to changing environments and to migrate efficiently within tissues.^10^ It was found that mechanical stimuli induce the migration of dendritic cells from tissues towards lymph nodes,^11^ where they eventually meet antigen-specific T-cells and promote an adaptive immune response. However, it has also been shown that the dense solid tumour microenvironment can drive T-cells into dysfunctionality,^12^ leaving cancer progression unaffected. Tumour cells on the other hand are believed to undergo a spontaneous switch to a fast ameboid migration type to adapt to this environment and eventually escape the primary tumor.^13^

To study the interaction of T-cells and APCs at the immunological synapse, supported lipid bilayers (SLBs) have emerged as an essential tool.^14–17^ They are exploited to mimic APC surfaces *in vitro* on various substrates for the following reasons: (i) synapses between SLBs and T-cells are oriented parallel to the substrate and enable high-contrast 2D imaging; (ii) the functionality of SLBs can be precisely adjusted by anchoring T-cell interacting proteins to the SLB; (iii) the SLB composition can tune diffusional properties of SLB-anchored proteins.

Technologies like micropipette aspiration, atomic force microscopy, and laser tweezers were utilized not only to study the role of force load and direction on TCR–pMHC binding kinetics^4,18^ but also to investigate membrane unfolding during cell deformation^19^ and to measure cell contractile forces during confinement,^7^ to mention just a few. While highly precise and sensitive to ultra-low forces, the throughput of these technologies is limited. To study the bulk behaviour of T-cells under mechanical stimuli, microfluidic devices have proven to be successful. They were used to measure molecular TCR–pMHC binding kinetics under tensile force,^20^ to study the migration and attachment of T-cells on dendritic cell monolayers at varying shear stress,^21^ and to investigate the behavior of cells compressed with varying force loads^22,23^ or confined to predefined heights.^24^ Existing microdevices (see ref. 25 for a comprehensive review) have been applied to study individual aspects of T-cell mechanobiology; however, to our knowledge, no device has been developed to mimic the T-cell microenvironment comprehensively by using artificial APCs.

**Fig. 1:**
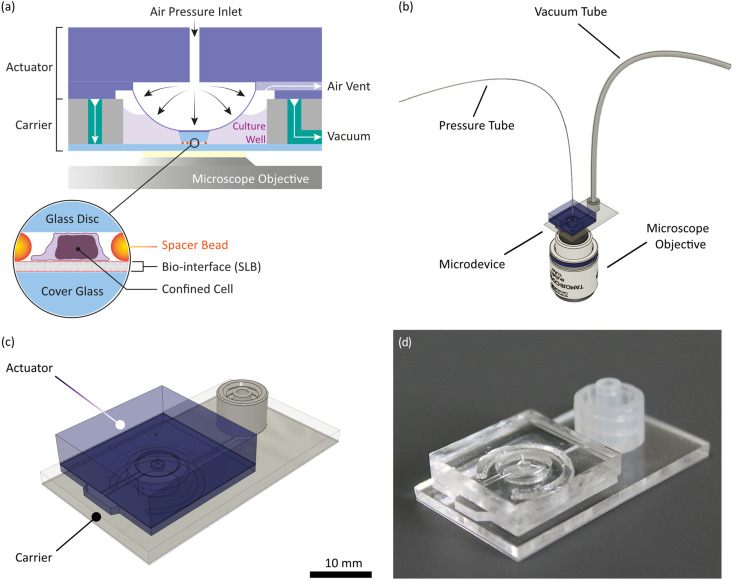
Microdevice design and function: (a) sketch of the microdevice during cell confinement and depiction of its individual functional components; (b) sketch of the microdevice within a microscope; (c) isometric view of the microdevice; (d) image of the microdevice.

In this study, we aim to go one step further by applying a modular microdevice that combines the mechano-environmental aspects of an APC mimicry with cell confinement. Consisting of actuator and carrier module (see Fig. 1), the assembled microdevice fits onto a standard microscope stage and enables the simultaneous characterization of tens to hundreds of cells. It is compatible with live-cell imaging and various epi-fluorescence and transmission light microscopy techniques, like total internal reflection fluorescence microscopy (TIRFM), interference reflection microscopy (IRM), confocal microscopy, or super-resolution imaging. As an actuating unit, we implemented an air pressure-driven PDMS membrane in the actuator module. The carrier acts as a cell culturing well, providing about 50 μL volume, a cover glass capable of hosting functional bio-interfaces and microchannels for vacuum fixation of the actuator. Microchannels on the actuator allow for venting off membrane-displaced air during membrane deflection. Cells are confined to preset heights between the glass surface of the carrier and a precision glass disc at the tip of the deflected actuator membrane, while microbeads act as spacers in between. These “spacer beads” offer high flexibility in choosing the desired confinement height and are applied by the user before an experiment. We chose glass as the confinement surface because, unlike PDMS, glass does not absorb small hydrophobic molecules and provides a chemically stable substrate.^26^ Both confining glass surfaces can be functionalized independently, offering the possibility to confine cells between two chemically distinct moieties, which is an advantage of our microdevice over closed microchannel designs. For operation, the carrier is connected to a vacuum source (MPXF5423050, Millipore) and the actuator to a pressure regulator (Flow EZ™ Push-Pull, Fluigent) *via* separate tubes (Fig. 1b). Due to the modular design, the modules can be assembled directly on the microscopy stage. This enables manual cell seeding onto the carrier before assembly of the actuator module. Consequently, cells can be imaged upon first contact with the bio-interface at desired cell densities. In addition, our microdevice is not a single-use item but can be cleaned, plasma- or UV-sterilized and reused (see ESI† and Fig. S10 and S11 for details). To validate our microdevice, we confined Jurkat T-cells on standard fibronectin-coated surfaces to 6 and 4 μm and confirmed confinement using 2D and 3D imaging. Compatibility with more sophisticated bio-interfaces was verified by preparing APC-mimicking SLBs with different compositions on the carrier glass surface and subsequent confining of primary murine T-cells.

## Experimental

### Microdevice fabrication

**Fig. 2:**
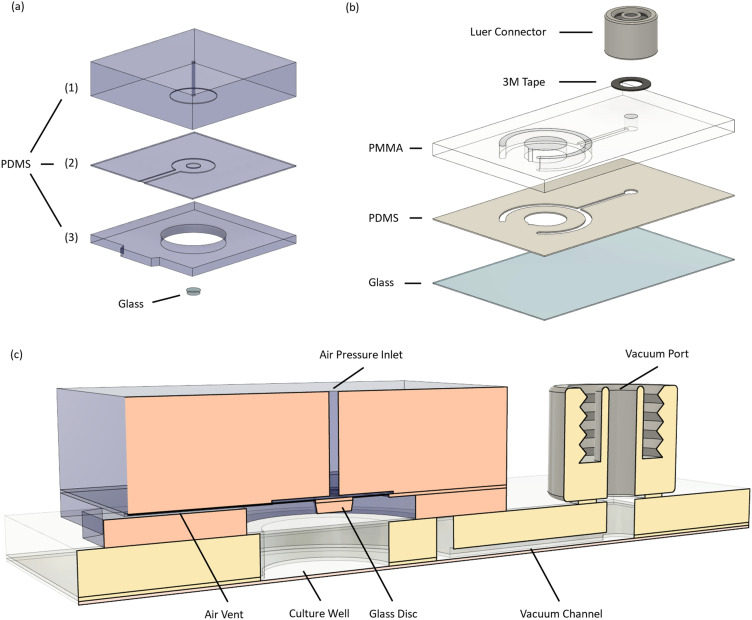
Composition of the microdevice: exploded view of the actuator (a) and carrier (b) showing their individual components and materials; (c) sectional view of the assembled microdevice showing the actuator (orange), the carrier (yellow) and additional feature annotations.

The microdevice and its modules are composed of different materials including polydimethylsiloxane (PDMS), polymethylmethacrylate (PMMA) and glass (Fig. 2). A detailed description of the fabrication process can be found in the ESI.† Briefly, all components were designed using CAD software (Autodesk Fusion 360, Autodesk). Micro-structured masters for PDMS casting were fabricated from silicon wafers using film masks (Micro Lithography Services Ltd) and standard photolithography techniques (SU-8 2015, SU-8 2075, micro resist technology). All PDMS parts were made from Sylgard 184 (Dow Corning) using a 10 : 1 mixing ratio (base : curing agent). The PDMS mixture was degassed under vacuum for 30 minutes after mixing and cured in the oven at 70 °C for 4 hours. Glass cover slides (#1.5, 24 mm × 40 mm, Carl Roth GmbH + Co. KG) and precision glass discs (*d* = 2 mm, *h* = 0.5 mm; #1659879, Schott) were sonicated in a cleaning agent (Hellmanex® III, Hellma), rinsed with distilled water and dried in the oven before usage. All PDMS parts were exposed to a 25 second O_2_ plasma activation (Femto, Diener Electronics) before bonding. Following each assembly step, parts were kept in the oven at 70 °C for 10 minutes to enhance bonding. PMMA components were processed with a laser cutter (Speedy 300, Trotec Laser GmbH). PMMA–PDMS bonding was performed with an adhesion promotor (DOWSIL™ 1200 OS Primer Clear, Dow). The adhesion promoter was spin-coated on plasma-activated PMMA and dried in the oven at 70 °C for 10 minutes before applying PDMS. PSA tape (VHB, 3M) was used to attach the Luer connectors (#11638, Qosina).

### Topography measurements

**Fig. 3:**
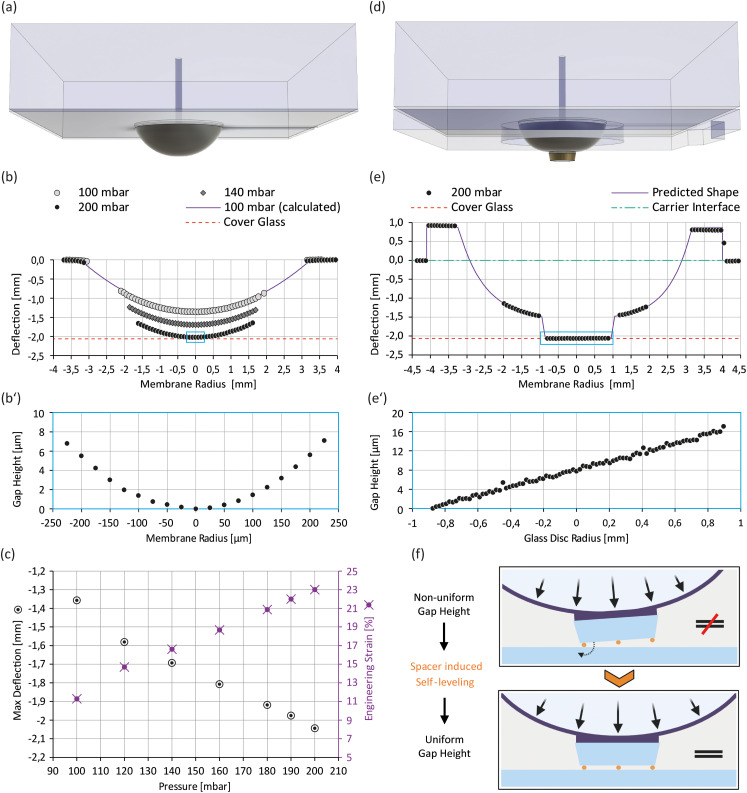
Validation of membrane deflection and gap size: (a) isometric view of the actuator module; (b) membrane deflection profiles at various pressures, including the calculated profile at 100 mbar (see the ESI† for additional information). The small box indicates the zoomed-in region shown in (b′); (b′) expected gap height between the membrane tip at 200 mbar and the cover glass surface; (c) max deflection (= deflection magnitude at membrane center) and calculated engineering strain as a function of pressure; (d) isometric view of the improved actuator module; (e) membrane deflection profile of the improved actuator module at 200 mbar. The small box indicates the zoomed-in region shown in (e′); (e′) expected gap height between the glass disc and the cover glass surface at 200 mbar; (f) principle of the spacer induced self-leveling on the improved actuator.

Actuator membrane topographies were measured with white light interferometry (Microprof MPR1201, Sensor 2100/0170-07, FRT). The actuators were placed membrane-side up into the instrument and pressurized before scanning the membrane surface. The actuator region representing the interfacing surface to the carrier module was used as a reference plane for global levelling. Topography profiles were extracted with SPIP software (6.0.3, Image Metrology) (see Fig. S3†) and analysed with MATLAB (version R2019b, MathWorks). Deflection and expected gap height plots (Fig. 3b, b′, e and e′) were generated from inverted topography data.

### Actuator preparation

#### Spacer bead immobilization

Spacer bead solutions were obtained from stock solution by dilution with distilled water (64070-15 (1 : 800); 64090-15 (1 : 400), Polysciences). Solutions were stored in the fridge and sonicated for 2 minutes before application. The actuator was plasma treated for 1 minute (glass disc side up; PDC-002, Harrick Plasma). 1 μl of bead solution was pipetted onto the glass disc and dried at room temperature for 10 minutes. Then, the actuator was placed in the oven at 70 °C for 20 minutes. Spacer bead distribution was checked under the microscope (see Fig. S2 and S10†).

#### Actuator recycling

The actuator was rinsed with ethanol, sonicated in cleaning agent (Hellmanex® III, Hellma), rinsed with distilled water, and dried in the oven (70 °C, 10 minutes). The glass disc surface was checked for residuals under the microscope. Cleaned actuators were stored in a dust-free atmosphere until usage.

### Carrier preparation

#### Fibronectin coating

The carrier was plasma-treated for 10 minutes (PDC-002, Harrick Plasma). 70 μl of fibronectin solution (F1141, Sigma Aldrich, Merck KGaA) was transferred into the culture well. The carrier was stored in a humidified incubator (37 °C, 5% CO_2_) for 1 hour. Finally, the fibronectin solution was aspirated, and the culture well was rinsed twice and refilled with imaging buffer (HBSS + 2% FBS (H8264 and F7524, both Sigma Aldrich)).

#### Supported lipid bilayers (SLBs)

A detailed workflow of SLB generation is described in the ESI.† Briefly, SLBs were made on plasma-cleaned glass surfaces and prepared from vesicles of 1-palmitoyl-2-oleoyl-*glycero*-3-phosphocholine (POPC, 850457C, Avanti Polar Lipids) and 1,2-dioleoyl-*sn-glycero*-3-[(*N*-(5-amino-1-carboxypentyl)iminodiacetic acid)succinyl] (nickel salt) (Ni-NTA-DGS, 790404P, Avanti Polar Lipids). B7-1 (50446-M08H, Sino Biological), ICAM-1 (50440-M08H, Sino Biological), and MMC-peptide loaded I-E^k^ (pMHC, prepared and labelled as described previously^27^) were bound to the SLBs *via* His-tags. Activating conditions (act): 2 ng I-Ek/MCC, 5 ng B7-1 and 5 ng ICAM-1; non-activating conditions (rest): 5 ng ICAM-1.

### Carrier recycling

The carrier was sonicated in cleaning agent (Hellmanex® III, Hellma), rinsed with distilled water, and dried in the oven (70 °C, 10 minutes). Cleaned carriers were stored under a dust-free atmosphere until usage.

### Jurkat T-cells

#### Culture

Jurkat T-cells (Clone E6.1, 88042803, Sigma Aldrich) were cultured in RPMI 1640 with l-glutamine (21875091, Gibco) supplemented with 10% FCS (F7524, Sigma Aldrich). Cells were split twice a week.

#### Membrane staining

Jurkat cells were stained with CellMask™ Deep Red Plasma Membrane Stain (C10046, Thermo Fisher Scientific). Briefly, 10^6^ cells were spun down (300*g* for 3 minutes), resuspended in 100 μl 1× CellMask™ working solution and incubated (37 °C, 5% CO_2_) for 10 minutes. Next, cells were washed and resuspended in 100 μl imaging buffer (HBSS + 2% FCS). Cells were stored on ice to avoid internalization of the probe and used within 30 minutes after staining.

### Mouse T-cells

#### Culture

T-cells were obtained from transgenic 5c.c7 mice as described previously.^28^ Briefly, T-cells were isolated from lymph nodes and stimulated with 2 μM HPLC-purified moth cytochrome *c* (MCC) peptide (ANERADLIAYLKQATK, Intavis Bioanalytical Instruments) in a T-cell medium (RPMI 1640 with l-glutamine (21875091, Gibco) containing 10% FCS (Biowest), 100 U mL^−1^ penicillin/streptomycin (15140130, Gibco), 2 mM glutamine (25030123, Gibco), 1 mM sodium pyruvate (11360070, Gibco), 1× non-essential amino acids (13114E, Lonza), and 50 μM mercaptoethanol (31350010, Gibco). The culture volume was doubled, and 100 U mL^−1^ IL-2 (#130-120-333, Miltenyi Biotec) was added on day 2. On days 3 and 5, T-cell cultures were expanded in a ratio of 1 : 1. On day 6, dead cells were removed by centrifugation through a Histopaque-1119 (#11191, Sigma Aldrich) cushion. T-cell experiments were performed on days 7–9 after initial stimulation.

#### Fura staining

About 10^6^ T-cells were incubated with 1 μg of Fura-2 AM dye (F1221, Thermo Scientific) for 20 minutes at room temperature. 5 ml of imaging buffer (HBSS + 2% FCS) were added to the cells, and the solution was centrifuged (300*g* for 3 minutes). The supernatant was removed, and cells were resuspended in 100 μl of imaging buffer. Cells were kept at room temperature and used within 90 minutes after staining.

#### Fixation and immunostaining

Prior to incubation with antibodies, cells were fixed (4% PFA in PBS, 20 min, RT), permeabilized (0.1% Triton X-100 in PBS, 10 min, RT), and blocked (0.5% BSA in PBS, 30 min, RT). Samples were then incubated overnight at 4 °C with the primary antibody targeting phosphorylated MAPK (Erk1/2) (#9101S, Cell Signaling) diluted 100-fold in PBS with 0.5% BSA. Cells were then labelled with secondary goat anti-rabbit antibody carrying Alexa Fluor 647 (#A21244, Thermo Fisher Scientific). The secondary antibody was diluted 200-fold in PBS containing 0.5% BSA and DAPI stain (#10236276001, Roche) at a concentration of 5 μg ml^−1^. Cells were washed with PBS and imaged after one hour of incubation. All washing steps were performed at least 5 times in a way that a small residual volume remained in the well to prevent cell detachment.

### Animal model and ethical compliance statement

Both male and female 5c.c7 αβ TCR-transgenic mice bred onto the B10.A background at 8–12 weeks old were randomly selected and sacrificed for isolation of T-cells from lymph nodes and spleen, which was evaluated by the ethics committees of the Medical University of Vienna and approved by the Federal Ministry of Science, Research and Economy, BMWFW (BMWFW-66.009/0378-WF/V/3b/2016). Animal husbandry, breeding and sacrifice of mice were performed in accordance with Austrian law (Federal Ministry for Science and Research, Vienna, Austria), the guidelines of the ethics committees of the Medical University of Vienna, and the guidelines of the Federation of Laboratory Animal Science Associations (FELASA), which match those of Animal Research: Reporting *in vivo* Experiments (ARRIVE).

### Imaging

#### Supported lipid bilayer imaging

Bilayers were imaged in total internal reflection (TIR) mode on an Axiovert 200 microscope (Zeiss). The microscope was equipped with a 642 nm diode laser (Oxxius), a 100× alpha-Plan Apochromat oil immersion objective (NA = 1.46, Zeiss) and an EMCCD camera (iXon DU 897-DV, Andor). A dichroic mirror (zt488/640rpc, Chroma) separated excitation and emission light. A 538/685 nm Bright Line® dual-bandpass filter (FF01-538/685-25, Semrock) served as an emission filter. Each bilayer was imaged for 200 frames with a frame rate of 40 Hz and an illumination time *t*_ill_ = 5 ms.

#### Live-cell imaging

Z-stacks of CellMask stained Jurkat cells were recorded using an Axiovert 200 M microscope (Zeiss) equipped with a 40× objective (Plan-NEOFLUAR 1.30 Oil, Zeiss) mounted on a PIFOC objective scanner (Physik Instrumente), and an iXon 897 EMCCD (Andor) camera. An image was taken every 0.5 μm using 640 nm laser excitation for 10 ms (i-Beam smart, Toptica) using appropriate fluorescence filters (650 long pass and HQ 680/30 M, Chroma). For calcium imaging of Fura-2 stained cells, excitation (340 and 380 nm, 45 ms and 5 ms illumination time, respectively) was performed using a Polychrome V light source (TILL Photonics). All hardware was controlled by Live Acquisition (version 2.12, FEI) software. Imaging was started right before T-cell seeding onto the bilayers. For confinement experiments, the recording was stopped after ten minutes, and an actuator was applied, which took about 30 seconds. Then a second recording was started at the same *X*/*Y* position, and the actuator pressure was slowly increased until the cells were fully confined, which took about 1–2 minutes. The second recording was stopped after 3–5 minutes.

For immunostaining experiments, cells were imaged on an inverted microscope (RVL2-K3, ECHO) in brightfield mode to check for proper cell confinement.

### Confocal imaging

Cells were imaged using a LSM780 Axio Observer confocal microscope system (Carl Zeiss). A 20× magnification objective (Plan-Apochromat 20×, NA 0.8, Carl Zeiss) was used. The two channels were acquired subsequently. The Alexa Fluor 647 signal was detected using 633 nm laser and collected between 638 and 756 nm range. DAPI was excited by the 405 nm laser and detected between 410 and 585 nm. The pixel size was 0.22 μm. Z-stacks were acquired using the same commercial setup with a 63× oil immersion objective (Plan-Apochromat 63×, NA 1.4 Oil DIC M27, Carl Zeiss). Immersol 518 F (Carl Zeiss) was used as an immersion oil. Laser lines and filters remained the same as when imaging with the 20× objective. Pixel size in this experiment was 0.07 μm and the distance between slices was set to 0.35 μm. Non-confined and confined T-cells were imaged at different regions on the same microdevice, *i.e.* outside and within the perimeter of the confining glass disc.

### Supported lipid bilayer characterization

SLB microscopy images were analysed in Python (sdt-python, 18.1).^29^ The antigen density of each bilayer was calculated by dividing the background corrected mean brightness per μm^2^ by the mean brightness of an individual fluorescently labelled antigen. The mean brightness per μm^2^ was determined from the first frame of each image series and averaged per SLB. During imaging, bleaching reduces the number of visible signals per area. Therefore, individual antigen molecules can be localized in later frames (sdt python, sdt.gui.locator, algorithm: daostorm_3d). To ensure good signal separation, we chose a density threshold of ∼0.09 μm^−2^ when analysing single molecule properties. Additionally, signals were filtered for size (0.75 px < signal size < 1.25 px). The mean single molecule brightness per SLB was calculated in an interval of 10 frames. The interval start was set individually for each image series to the first frame that met the density threshold. To assess the antigen mobility, the localizations were tracked with trackpy.link^30^ (tracking radius: 6). We selected signals from two low-density (0.03 ng pMHC) activating bilayers within a chosen area that is illuminated approx. evenly by the Gaussian-shaped laser profile (see box in Fig. 5a). All frames were used for the analysis. Diffusion constants were calculated from all trajectories with a minimum length of 5 steps using sdt.motion.msd.

### Cell data processing

#### Fura-2 imaging data

Raw data image stacks containing Fura-2 emission signals from 340 and 380 nm excitation (“340 and 380 nm emission images”) were processed with ImageJ (1.53t). Fura-2 sum image stacks were generated by summing of 340 and 380 nm emission images (Calculator Plus: https://imagej.net/ij/plugins/calculator-plus.html), while Fura-2 ratio image stacks were generated by division of 340 and 380 nm emission images after background subtraction (Ratio Plus: https://imagej.net/ij/plugins/ratio-plus.html). T-cells were segmented and tracked using the Fura-2 sum image stacks with CellProfiler^31^ (4.2.1) (https://cellprofiler.org) running a custom-made analysis pipeline. The tracking data were processed and further analysed in MATLAB (version R2019b, MathWorks). The term “sectional cell area” corresponds to the segmented area of a single cell, whereas the “Ca^2+^ ratio” corresponds to the mean Fura-2 ratio signal in the segmented cell area. “Sectional cell area” and “Ca^2+^ ratio” were plotted for each tracked cell (grey lines). The mean values averaged over all cells are shown in the respective plots (bold black and coloured lines) and dotted vertical lines indicate the data joining point of non-confined and confined data sets. For statistical analysis we compared the mean values before and after confinement within defined time windows (bold coloured lines matching boxplot colours). We choose time windows with minimal time gap between unconfined and fully confined states. The detailed cell data processing workflow is described in the ESI.†

Cell motility analysis was performed using the tracked cell location centres, and diffusion constant *D* and global movement speed *V* were obtained from mean-square-displacement (MSD) analysis. Specifically, all trajectories from all cells under a given condition (rest^(−)/(+)^, act^(−)/(+)^) were pooled and the MSD *versus t*_lag_ plot was determined.^32^ Data were fitted using a model that accounts for both diffusion and directed motion:MSD = 4*Dt* + *V*^2^*t*^2^ + 4PA^2^,where *D* is the diffusion constant, *V* is the global movement speed, and PA represents positional accuracy. The average local velocity and the directional persistence for individual cells under a given condition (rest^(−)/(+)^, act^(−)/(+)^) were calculated as described in Park *et al.*^33^

#### pErk1/2 imaging data

Confocal images were processed with CellProfiler (4.2.1) running a custom-made analysis pipeline. “Sectional nucleus areas” were determined by DAPI signal segmentation and “sectional cell areas” by pErk1/2 signal segmentation. The mean pErk1/2 signal (pErk1/2 mean intensity) was measured within the pErk1/2 segmented areas. The segmentation data were processed and plotted with MATLAB (version R2019b, MathWorks).

### Statistical analysis

Pairwise two-tailed Student's *t*-tests assuming unequal variances were used to check for statistical difference between two groups of cells (*i.e.* rest^(−)^ and rest^(+)^). Student's *t*-tests were performed in Excel using either the Data Analysis ToolPak (https://www.statisticshowto.com/excel-data-analysis-toolpak/) or the Real Statistics Resource Pack (https://real-statistics.com/free-download/real-statistics-resource-pack/). To check for significant differences between multiple groups, we used ANOVA and Games Howell multiple comparison test (https://real-statistics.com/one-way-analysis-of-variance-anova/unplanned-comparisons/games-howell-test-2/). We used *p* = 0.05 as the significance level in all tests. If not stated otherwise, all values were reported as mean (*m*) ± standard deviation (*σ*). All boxplots feature notches which, if not overlapping, indicate a difference of median values with 95% confidence.^34^ Values more than 1.5 times the interquartile range away from the bottom or top of the box were drawn as outliers.

## Results

### Actuator design optimization

The deflectable actuator membrane is a crucial component of our microdevice that features a nonstructured PDMS membrane with a thickness of 160 μm. To squeeze cells on the carrier cover glass surface it must be deflectable to a minimum distance of about 2 mm which represents the well depth of the carrier module (see Fig. S3g†). When measuring the deflection profile of the actuator membranes at various pressures, we found that a working pressure of 200 mbar is sufficient to reach this deflection threshold (Fig. 3b). Moreover, we observed a roughly linear relation between the deflection magnitude at the membrane centre (max deflection) and the applied pressure (Fig. 3c). Given this linear behaviour and our pressure controller's resolution of 0.1 mbar, we expect a theoretical resolution of 0.65 μm/0.1 mbar for tuning the gap size in our microdevice. However, actuator characteristics depend mostly on the mechanical properties of the PDMS membrane. Hence, working pressure and height resolution may vary between actuator units due to prototyping artefacts. To determine the mechanical properties of our membrane, we fitted the deflection profiles with 12th-order parabolas (see Fig. S4†).

From the parabola arc lengths, we could calculate the membrane's engineering strain at different working pressures (see Fig. 3c and the ESI†). At the working pressure of 200 mbar, the membrane is stretched by about 23% perpendicular to its perimeter. Based on the small initial thickness of our membrane and the calculated engineering strain at working pressure, we expect its mechanical properties (*i.e.* Young's modulus and Poisson ratio) to change (I) during the process of membrane deflection (thickness/strain dependence^35^) and (II) with time at constant working pressures (time dependence^36^). This implies that the membrane is not well suited to squeeze cells under constant force loads or to confine them to defined heights, as both force and confinement height would change with time. More importantly, the observed membrane curvature (see Fig. 3b′) would not allow uniform confinement of cells but instead results in a height gradient, and hence the actuator does not meet our requirement for uniform cell confinement. We therefore adapted its design by adding a glass disc at the membrane centre that stiffens the membrane tip and provides a defined confinement surface (Fig. 3d). Fig. 3e shows the cross-sectional profile of the deflected membrane in our improved actuator module at 200 mbar, while Fig. 3e′ indicates the expected gap height in the assembled microdevice based on topography data (see Fig. S3†). Fig. 3e′ demonstrates that perfect coplanarity between both confining surfaces, and hence uniform cell confinement, is not guaranteed by design, a circumstance linked to imperfections in materials and the limitations of prototyping processes. It thereby highlights the need of spacer features, which render the coplanarity of confinement surfaces independent of these manufacturing defects. To overcome this limitation, we immobilize spacer beads on the glass disc and deflect the membrane to an endpoint (>200 mbar), which promotes self-levelling of the glass disc (Fig. 3f). These spacer beads preset the confinement height and hence decouple the membrane's mechanical properties from the confinement process. As a result, a uniform confinement height is ensured at preset confinement heights. To compensate for the glass disc's additional height and improve air venting, we added a 3rd PDMS layer at the bottom of the actuator (Fig. 2a and 3d).

### Microdevice validation

#### Cell confinement

**Fig. 4:**
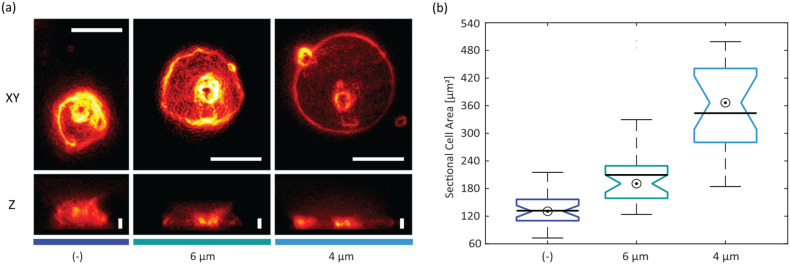
Cell confinement and SLB: (a) wide-field fluorescence images of Jurkat T-cells on fibronectin-coated surfaces: *xy* and *z* projection of non-confined (−) and confined (6 μm and 4 μm); scalebars: *XY*, 10 μm; *Z*, 4 μm; (b) sectional cell areas of non-confined (−) and confined (6 μm and 4 μm) Jurkat T-cells (bullseye: median; line: mean; + outlier; *N*_(−)_ = 32, *N*_6μm_ = 34, *N*_4μm_ = 19 cells). All median values are significantly different on a 95% confidence level (non-overlapping boxplot notches).

Jurkat T-cells were stained with CellMask™ Deep Red and seeded onto a fibronectin-coated carrier glass surface. The actuator was mounted onto the carrier, and cells were confined 10 minutes after seeding. Z-stacks of cells were recorded *via* wide-field fluorescence microscopy to validate the microdevice's confinement functionality and the confinement heights preset by the spacer beads. Fig. 4a shows the *XY* and *Z* projections of the stained Jurkat T-cells in non-confined (−) and confined states using actuator glass discs covered with 6 μm- or 4 μm-sized spacer beads, respectively. The sectional cell areas – determined from *XY* projections – relate to the bead size and differ significantly among the three test conditions (see Fig. 4b). In agreement, *Z* projections show a reduction in cell height upon confinement, which compares to the diameter of spacer beads used in the experiments (Fig. 4a). Footage recorded at the edge of the confining glass disc shows that the microdevice can also be used to study non-confined and confined cells next to each other (see Fig. S5†).

### Device compatibility with functionalized bio-interfaces

**Fig. 5:**
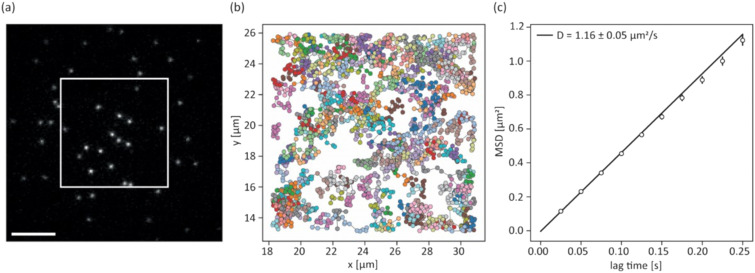
Supported lipid bilayer characterization: (a) TIRF image of pMHC-AF647 anchored to a supported lipid bilayer (scalebar: 5 μm). Signals within the box were taken for single-molecule analysis; (b) single-molecule trajectories of pMHC-AF647 molecules on the SLB, each individual track is shown in a separate color; (c) MSD–lag time plot of tracked pMHC-AF647 (*N* = 222 molecules from two SLBs) shows free Brownian motion of molecules.

Studying cell–cell interactions is often limited by the arbitrary 3D orientation of their emerging synapse. For high-contrast imaging using total internal reflection fluorescence (TIRF) microscopy, a 2D synapse can be enforced by mimicking one interaction partner using an SLB bio-interface. In addition, the composition of such bio-interfaces can be precisely tuned to study the role of particular proteins within the synapse.^27,37,38^ To demonstrate the device compatibility with such bio-interfaces, the carrier glass surface was coated with an APC surface mimicry composed of a lipid bilayer functionalized with His-tagged proteins for adhesion (ICAM-1), co-stimulation (B7-1) and activation (fluorescently labelled pMHC). Single-molecule fluorescence microscopy yielded high-contrast diffraction-limited signals of single pMHC molecules under TIRF illumination (Fig. 5a). Single-molecule tracking of pMHC verified an intact lipid bilayer membrane with >98% of pMHC molecules diffusing freely with *D* = 1.16 μm^2^ s^−1^ (Fig. 5 and S6†).

### Microdevice application

#### T-cell Ca^2+^ signalling upon confinement

**Fig. 6:**
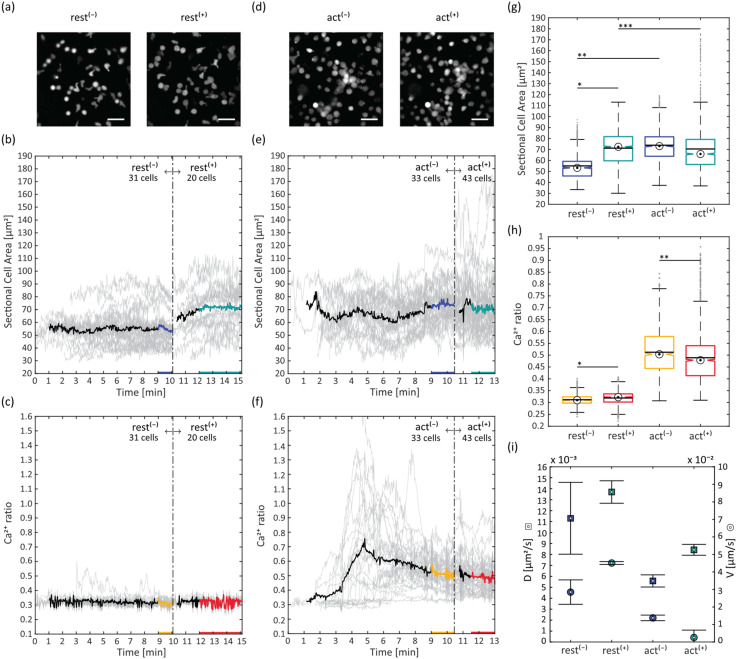
T-cell confinement to 6 μm on SLBs: (a) Fura-2 sum image of T-cells on resting SLB, unconfined (rest^(−)^) and confined (rest^(+)^) (scalebar: 25 μm); (b) sectional cell area on non-activating SLB, before (rest^(−)^) and after confinement (rest^(+)^), for individual cells (gray lines) or averaged over all cells (bold black, blue and green line); (c) Ca^2+^ ratio on non-activating SLB, before (rest^(−)^) and after confinement (rest^(+)^), for individual cells (gray lines) or averaged over all cells (bold black, yellow and red line); (d) Fura-2 sum image of T-cells on activating SLB, unconfined (act^(−)^) and confined (act^(+)^) (scalebar: 25 μm); (e) sectional cell area on activating SLB, before (act^(−)^) and after confinement (act^(+)^), for individual cells (gray lines) or averaged over all cells ((bold black, blue and green line)); (f) Ca^2+^ ratio on activating SLB, before (act^(−)^) and after confinement (act^(+)^), for individual cells (gray lines) or averaged over all cells ((bold black, yellow and red line)); (g) sectional cell area boxplots (bullseye: median; line: mean), */** are significantly different (*p* < 0.05), *** is not significantly different (*p* > 0.05); only the blue and green colored data from (b) and (e) were considered; (h) Ca^2+^ ratio boxplots (bullseye: median; line: mean), */** are significantly different (*p* < 0.05); only the yellow and red colored data from (c) and (f) were considered; (i) diffusion coefficient *D* and global movement speed *V* from MSD analysis (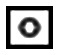 and 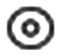 are mean values, error bars represent s.d. and were determined *via* bootstrapping).

Next, we aimed to combine bio-interface compatibility with the confinement of primary murine T-cells stained with the ratiometric calcium indicator Fura-2 and seeded onto carrier glass supported lipid bilayers functionalized with His-tagged proteins (non-activating bilayer (rest): ICAM-1 only, activating bilayer (act): B7-1, ICAM-1 and pMHC). The Fura-2 signal upon excitation with 340 and 380 nm light was recorded on a time course of 13–15 minutes. Ten minutes after seeding, an actuator covered with 6 μm-sized spacer beads was applied onto the carrier module and T-cells were confined. Sectional cell areas and Ca^2+^ ratios were determined for individual cells and mean values were compared before and after confinement. T-cells seeded onto the non-activating bilayer showed an increased mean sectional cell area upon confinement to 6 μm (Fig. 6a, b and g). This was even more pronounced when confining to 4 μm (see Fig. S7†). However, we observed frequent events of cell ruptures at 4 μm, interfering with cell segmentation and tracking. Therefore, we continued with 6 μm confinement height only. Cells seeded onto the activating bilayer spread out after seeding and showed significantly higher mean sectional cell areas even before confinement compared to the non-activating bilayer; however, their mean sectional cell areas did not change considerably upon confinement to 6 μm, despite individual cells showing increased values (Fig. 6d, e and g). As expected, T-cells on the activating bilayer containing antigenic pMHC displayed typical Ca^2+^ ratio activation curves (Fig. 6f and h), with the number of responsive cells depending on the antigen density on the supported lipid bilayer (Fig. S8e–h†). On the non-activating bilayer, the mean Ca^2+^ ratio was significantly lower (Fig. 6c and h). Subsequent confinement on activating SLBs did not lead to an additional increase of Ca^2+^ signalling. However, we observed small but significant increases in intracellular Ca^2+^ levels in resting T-cells (Fig. 6h). As an additional parameter, we determined the mean square displacement of moving cells from their trajectories and calculated the diffusion coefficient *D* and global movement speed *V* (Fig. 6i). For T-cells on the non-activating bilayer, these motility parameters were higher compared to the activating bilayer condition. Under additional confinement, resting T-cells showed an increased motility, while activated T-cells had a reduced global movement speed but an increased diffusion constant.

A similar trend was found for the average local velocity (Fig. S9†), while the directional persistence increased under confinement for both resting and activated cells.

### Erk1/2 signalling upon T-cell confinement

**Fig. 7:**
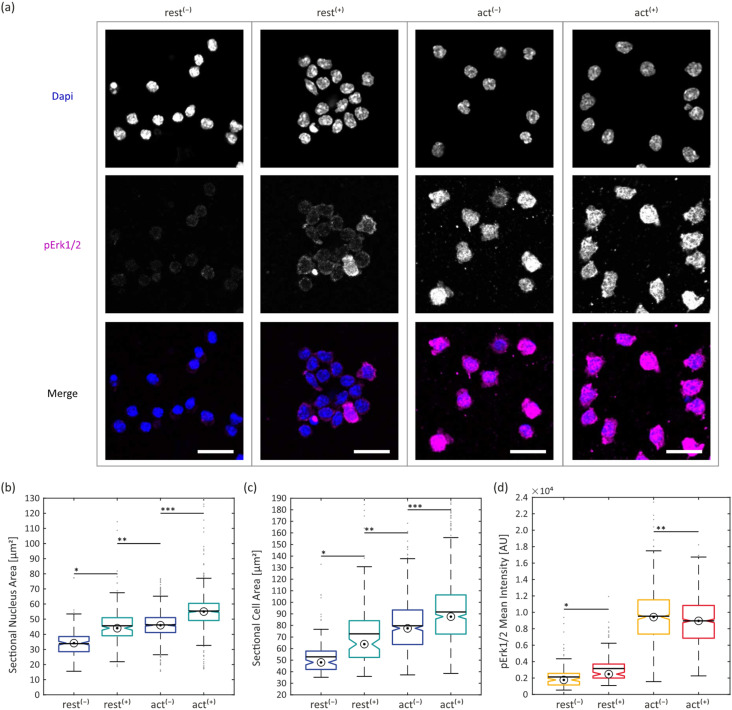
Immunostaining: (a) fluorescence confocal images of immunostained T-cells; top row: DAPI, middle row: pErk1/2, bottom row: DAPI and pErk1/2 merged (same contrast within rows; scalebar: 20 μm); (b) sectional nucleus area on non-activating (rest) and activating (act) SLB, before (−) and after (+) confinement, (bullseye: median; line: mean; *N*(rest^(−)^) = 174; *N*(rest^(+)^) = 204; *N*(act^(−)^) = 677; *N*(act^(+)^) = 1003 cells), */*** are significantly different (*p* < 0.05), ** is not significantly different (*p* > 0.05); (c) sectional cell area on non-activating (rest) and activating (act) SLB, before (−) and after (+) confinement, (bullseye: median; line: mean; *N*(rest^(−)^) = 133; *N*(rest^(+)^) = 132; *N*(act^(−)^) = 544; *N*(act^(+)^) = 731 cells), */**/*** are significantly different (*p* < 0.05); (d) pErk1/2 mean intensity on non-activating (rest) and activating (act) SLB, before (−) and after (+) confinement, (bullseye: median; line: mean; *N*(rest^(−)^) = 133; *N*(rest^(+)^) = 132; *N*(act^(−)^) = 544; *N*(act^(+)^) = 731 cells), */** are significantly different (*p* < 0.05).

To study the effect of confinement on TCR signalling more comprehensively, we aimed to quantify its influence on an alternative TCR signalling pathway. We therefore targeted the RASGRP1–RAS–ERK1/2–AP1 pathway, more specifically the MAPK extracellular signal-regulated kinase-1 and 2 (ERK1/2) within the RAS-MAPK cascade which has an important role in controlling T-cell development, differentiation, and TCR-induced signal strength.^39^ T-cells were seeded onto carrier glass supported lipid bilayers functionalized with His-tagged proteins (non-activating bilayer (rest): ICAM-1 only, activating bilayer (act): B7-1, ICAM-1 and pMHC). Ten minutes after seeding, an actuator covered with 6 μm-sized spacer beads was applied onto the carrier module and cells were confined for 5 minutes. Afterwards, confinement was released, the actuator was removed, and cells were fixed and immunostained for phosphorylated Erk1/2 (pErk1/2). In addition, DAPI staining was applied as a reference marker. In the non-confined conditions, cells were kept on the bilayer for the same total time before fixation and immunostaining. All cell samples were imaged by confocal microscopy. Sectional nucleus areas and sectional cell areas were determined by segmentation of DAPI and pErk1/2 signals, respectively. The pErk1/2 intensities were determined for individual cells within segmented areas. T-cells on activating bilayers showed significantly higher pErk1/2 signals compared to the resting conditions, indicating effective TCR signalling in the presence of the specific antigen (Fig. 7a and d). Interestingly, confinement led to an increase of pErk1/2 signal in the resting state (Fig. 7d). In the activating state, we could observe a slight decrease of pErk1/2 signal. This trend was also observed earlier for the Ca^2+^ ratios (see Fig. 6h). The determined mean sectional cell areas were in line with values obtained earlier from Fura-2 signal segmentation (Fig. 6g and 7c). T-cells on activating bilayers spread out more compared to cells on resting bilayers. Confinement increased the mean sectional cell areas additionally in all conditions. Moreover, we could observe and quantify compression of cell nuclei upon confinement in both resting and activating conditions. Activated cells displayed a larger mean sectional nucleus area compared to resting ones, even before confinement (see Fig. 7b).

## Discussion

Actuator characterization revealed that the original design could not ensure uniform cell confinement, prompting us to optimize it for coplanarity using spacer-induced self-levelling. This approach decouples membrane mechanics from confinement control, compensates for prototyping imperfections, and eliminates the need for sensors or precision pressure controllers. While this results in fixed (preset) confinement heights, smooth transitions remain possible, though without guaranteed coplanarity of the confining surfaces. The modular microdevice design allows sequential changes in confinement height by swapping actuator modules. The use of spacers, as seen in other microdevices (*e.g.* in the form of PDMS micro-pillars^24,40^), reflects the importance of controlled confinement, with our design leveraging a rigid glass disc to enable random placement of spacer beads.

With the help of our developed microdevice we could show that T-cell outer dimensions and T-cell nuclei sizes are affected not only by confinement but also by SLB composition. With increasing antigen density, T-cells continue to spread out onto the flat SLB, which could be quantified by measuring an increase in their sectional cell and sectional nucleus areas. Similarly, confinement of T-cells to 6 μm increases these metrics. Notably, at the same confinement height, activated T-cells showed increased areas compared to resting T-cells. This observation implies that cellular and nuclear volume are increased upon T-cell activation, a finding that was previously also confirmed by others.^41^ Using micropipette aspiration techniques, Waugh *et al.*^41^ measured an ∼3-fold increase in T-cell volume upon activation, which aligns well with our sectional cell area data comparing resting with activating conditions. This increase in cellular volume is driven by ion channel activity, osmotic shifts, cytoskeletal remodelling and metabolic upregulation. Indeed, cell volume regulation was found to be essential for optimal T-cell function with volume-regulated anion channels (VRACs) playing a critical role in T-cell activation.^42^ Xu *et al.* identified a decompaction of chromatin in association with a disruption of the nuclear envelope during T lymphocyte activation.^43^ This was coupled with an overall increased nucleus size, which is in line with our findings of increased sectional nucleus areas in activated T-cells.

We observed effective T-cell triggering on activating *versus* resting SLBs by increased intracellular Ca^2+^ levels and Erk1/2 phosphorylation. Confinement on activating SLBs did not lead to an additional increase of activation signals in both assays. However, we observed small but significant increases in intracellular Ca^2+^ as well as pErk1/2 levels in resting T-cells upon confinement. The observed Ca^2+^ increase may be attributed to Ca^2+^-permeable channels gated by confinement-induced plasma membrane deformation and stretching. Different Ca^2+^-specific mechanosensitive channels have been described in the literature, *i.e.* TRPV2 in the Jurkat cells^44^ and Piezo1 in primary human T-cells.^45^ Liu *et al.* state that a Piezo1-driven Ca^2+^ influx drives cytoskeletal rearrangements which aid TCR signalling and hence improve T-cell activation.

Also, Erk1/2 signalling upon mechanical stimulation has been observed before,^46^*i.e.* under compressive stimulation in epithelia cells.^47^ Furthermore, Baschieri *et al.* reported transient Erk1/2 activation and translocation from the cytoplasm into the nucleus in HeLa cells confined under an agarose plug.^48^

Despite a measurable effect of confinement, the Ca^2+^ and pErk1/2 signals of resting T-cells under confinement were still significantly lower as compared to activated T-cells. This finding seems logical, as T-cell activation should not be triggered solely by mechanical cues present in their microenvironment but should be the result of the highly sensitive antigen recognition process within the immunological synapse.

Mechanical stimuli do however support effective T-cell activation and aid in regulating the immune response.^45^

We could further observe an increased motility in resting T-cells under confinement, which aligns with results from Park *et al.*,^33^ who described velocity and persistence increases, and morphological changes in confined T-cells. They further provided evidence that microtubule dynamics are the critical cytoskeletal component involved in these processes. Confinement-induced cellular reflexes^7,8^ were previously also shown for other cell types like mesenchymal cells,^13^ immature mouse dendritic cells^7^ or early zebrafish progenitor cells.^49^

Activated T-cells showed reduced motility as compared to resting ones in our experiments. Beemiller *et al.* also observed a decrease in motility with increasing antigen concentration in OT-1 T-cells seeded on SLBs.^50^ Their work also suggests that T-cells coordinate TCR signalling with motility, especially in motile synapses. Additional confinement reduced the global movement speed of activated T-cells further, while we observed an increase of diffusion constant *D*. We speculate, that the observed change in global movement speed is a combined effect of increased integrin activity in activated T-cells and confinement-induced increase of interaction area between T-cell and ICAM-1 bearing SLBs. The rise in diffusion constant upon confinement may be attributed to increased cell sectional areas in combination with changes in T-cell morphology.

## Conclusion and outlook

The natural T-cell microenvironment is complex and governed by diverse mechanical cues that aid T-cell-specific functions in the adaptive immune system. Mimicking this microenvironment is essential to study T-cell mechanoimmunology *in vitro*. Microfluidic devices have been used successfully to mimic aspects of natural cell environments,^25^ but no device has been built yet that comprehensively mimics the T-cell mechanoenvironment. We aimed to combine the mechanoenvironmental aspects of an APC mimicry with compression and developed a microdevice capable of precise cell confinement on functional bio-interfaces. To enhance user-friendliness, we developed a microdevice with an innovative modular design that enables:

(i) Independent functionalization of its glass confinement surfaces with distinct bio-interfaces.

(ii) Presetting an accurate confinement height before an experiment using “spacer beads”, thereby avoiding the need for microfabricated PDMS pillars.^22,24^

(iii) A uniform confinement gap achieved through spacer bead-induced self-levelling between stiff glass surfaces.

(iv) Manual cell seeding of desired cell densities and live imaging of first cell bio-interface contacts before confinement.

(v) Assembly of the microdevice directly on the microscope and allowing for different combinations of actuator and carrier modules.

(vi) Use of standard and cost-effective lab equipment by eliminating the need for precision pressure or vacuum sensors and controllers.^22,24^

(vii) Cleaning, sterilization and reuse (see the ESI† and Fig. S10 and S11 for details) of individual modules and their confining glass surfaces – a significant advantage over closed microfluidic chips and microdevices using PDMS as a confining surface.^22,25^

We showcased our microdevice by confining primary mouse T-cells on APC mimicking SLBs. Live-cell imaging and a customized analysis pipeline enabled us to quantify T-cell mechanotransduction by tracking changes in cell morphology, intracellular calcium levels and migration-associated metrics before, during and after confinement to preset heights. Moreover, we proofed our microdevice compatibility with immunostaining and investigated the effects of mechanical stimulation and SLB composition on intracellular TCR associated signalling events. While our study focused on the interaction of T-cells with a surrogate APC generated on the glass substrate of the carrier, future applications could leverage the glass disc on the actuator to create a second functional surface. This could involve coatings with ECM components of the natural T-cell microenvironment, like fibronectin or collagen, which have been used to study T-cell migration under confinement.^10,51^ Other surface modifications may involve silane-based functionalization, for presentation of specific ligands, or hydrogel coatings to tune the stiffness of the confining surfaces. Moreover, this approach would enable the study of high cell densities, such as those found in immune organs like the spleen or lymph nodes,^52^ under mechanical confinement. Future design variants of the carrier module may include flow cell elements for media exchange or generation of chemical gradients. The actuator module may be utilized for timed, transient and repeated cell confinement to preset heights. Moreover, its membrane actuation could be exploited to apply shear forces onto surface-attached cells by means of controlled deflection associated fluid displacement. Despite being developed to study T-cells, our microdevice may also be considered as a platform for mechanical stimulation studies of other cell types in the presence of various bio-interfaces. Given the size of the carriers' culture well, our microdevice could also be exploited to study spheroids, organoids or even tissue samples under mechanical loads.

## Data availability

Data for this article, including photomask designs, figure data, cell tracking data, and data processing tools including source codes are available at TU Wien data repository at https://doi.org/10.48436/gx08m-d2y86.

## Author contributions

Conceptualization: C. T., M. Br., G. S. Data curation: C. T., C. K., B. K. Formal analysis: C. T., C. K. Funding acquisition: G. S., M. Br. Investigation: C. T., C. K., B. K., N. S., M. Bi, M. Br. Methodology: C. T. Project administration: C. T., M. Br. Resources: G. S. Software: C. T., C. K. Supervision: M. Br. Validation: C. T., M. Br. Visualization: C. T., C. K. Writing – original draft: C. T., M. Br. Writing – review & editing: C. T., M. Br.

## Conflicts of interest

There are no conflicts to declare.

## Supplementary Material

LC-025-D5LC00248F-s001
