# Supplementary material for: Microdevice for confinement of T-cells on functionalized bio-interfaces
Source: Lab Chip. 2025 Apr 22;25(11):2654–68. doi: 10.1039/d5lc00248f (PMC12041909; doi:10.1039/d5lc00248f)
Supplement: LC-025-D5LC00248F-s001 [file LC-025-D5LC00248F-s001.pdf]

Supplementary Information (SI) for Lab on a Chip.
This journal is © The Royal Society of Chemistry 2025

Electronic supplementary information (ESI)

Microdevice fabrication – Detailed workflow

The Actuator (see Fig. 2a) consists of three PDMS parts and a precision glass disc (d = 2 mm, h = 0.5 mm; #1659879, Schott). PDMS parts (1) and (2) were casted from two SU8 master which were fabricated using standard photolithography techniques (SU-8 2015, SU-8 2075, micro resist technology). Film masks were purchased from Micro Lithography Services Ltd. Each PDMS cast contained ten replicas of each part. Part (1) was designed to have a cylindrical notch at its bottom (d = 6.5 mm; h = 120 µm). An additional clearance hole was punched-out at the notch centre using a 0.2 mm biopsy-puncher. For Part (2), the PDMS was spin-coated onto the SU8 master to yield a layer height of 160 µm. It features a ring-shaped notch (d_{out} = 6 mm; d_{in} = 2 mm; h = 120 µm) connected to a straight channel at the bottom. Part (3) was laser-cut from a PDMS sheet with a thickness of 1 mm and has a central through hole (d = 7mm). All PDMS parts were manually aligned and bonded after 25 seconds of plasma activation (O_2 plasma; Femto, Diener electronics). After each bonding step the semi-finished products were kept in the oven at 70 °C for 10 minutes to enhance bonding. The pre-cleaned glass disc was plasma cleaned for 5 minutes and bonded to the cylindrical platform in the membrane centre after 25 seconds PDMS plasma activation, yielding the finished Actuator.

The Carrier (Fig. 2b) was made from PMMA, PDMS and a glass cover slide (#630-1844, VWR). A laser cut PMMA disc (d = 6 inch, h = 2 mm) was plasma-activated for 25 seconds, spin-coated with adhesion promotor (DOWSIL™ 1200 OS Primer Clear, Dow) and dried in the oven at 70 °C for 10 minutes. A 175 µm thick PDMS layer was then spin-coated on top. After oven curing, nine rectangular pieces (24 x 40 mm²) with additional features were cut out from the disc with a laser cutter. Each piece had two through holes, one for the culture well (d = 6 mm) and one for the vacuum port (d = 2 mm). Additionally, it had a C-shaped cut-out connected with the vacuum port via a 200 µm deep laser-engraved ditch on the PDMS side. All pieces were firmly cleansed to remove any deposits from laser processing. A pre-cleaned glass microscope slide was plasma cleaned for 5 minutes and bonded onto the PDMS side of each piece, following a 25 second PDMS plasma activation. Carrier assembly was completed by gluing a Luer connector (#11638, Qosina) onto the vacuum inlet hole using a laser-cut double sided tape (VHB, 3M).

Volume extenders (Fig. S1), used for lipid bilayer preparation, were produced in a similar fashion to the Carriers. These PMMA-PDMS composites were made from a 5 mm PMMA and a 2 mm PDMS layer. The laser-cut pieces (22 x 22 mm²) have through holes with a diameter of 8 mm. When attached onto the carrier, the volume extender increases the volume of the cell culture well by about 350 µl.

Actuator Membrane Modelling

The deflectable Actuator membrane is a key component of our microdevice that features a flat PDMS membrane with a thickness of 160 µm. We analysed the membrane's stretch performance by scanning the Actuator surface at varying pressures using white light interferometry. From the topography data we extracted the cross-sectional profiles of the deflected membranes (see Fig. 3b and Fig. S3) and modelled these profiles using Hencky`s[1] equation as described by Zarghamee et al[2]. The equation describes the maximal deflection h_{max} as a function of membrane radius *r*, membrane thickness *t*, Youngs modulus *E* and the pressure difference inside and outside of the membrane $\Delta p = p_{in-}p_{out}$:

$$h_{max} = r * \alpha * \left[\frac{r}{E*t} * (\Delta p)\right]^{1/3} \quad \text{(I)}$$

The dimensionless coefficient α is a function of the Poisson ration v. For a circular membrane it is calculated as follows[2]:

$$\alpha = 0.72 * (1 - v^2)^{1/3} \quad \text{(II)}$$

For small deflections we consider the membrane to have a 2nd order parabolic shape:

$$h = a * x^2 + b * x + c \quad \text{(III)}$$

With $h = h_{max}\ at\ x = 0$ and $h = 0\ for\ x = |r|$ it follows that:

$$a = -\frac{h_{max}}{r^2}, b = 0, and\ c = h_{max}$$

The negative coefficient *a* reflects the membrane's downward deflection into the Carrier well. For material properties data we refer to the work of Dogru et al.[3], who studied the viscoelastic properties of thin PDMS films experimentally using a non-contact measurement technique. Notably, the mechanical properties of thin PDMS films and bulk PDMS differ drastically. Liu et al.[4] discovered that mechanical strength and Youngs modulus become thickness dependent below a threshold thickness of about 200 µm. For thinner PDMS layers, the reordering of polymer chains upon strain dominates the mechanical properties. Moreover, the stress-strain curves were found to behave non-linear for films in this thickness regime. Further, Dogru et al.[3] discovered a time dependent relaxation of Poisson ratio and Youngs modulus. After stretching a 155 µm thick PDMS membrane unidirectionally by a 10% step stretch, they observed a decrease in Youngs modulus by ~3.4%[3] and Poisson ratio by ~2.1%[3] over a period of 20 minutes. The thickness of the PDMS membrane in our Actuator is comparable to the PDMS film used in their study. Hence, we expect changing mechanical properties (i.e. Youngs modulus and Poisson ratio) with strain and time as we deflect our Actuator's membrane.

For modelling the membrane deflection, we approximate the 10% step stretch of S.Dogru et al.[3] by defining our engineering strain *e* as

$$e = (s - d)/d \quad \text{(IV)}$$

Where *s* is the is the arc length of the membrane's parabolic cross-sectional profile under deflection and *d* the initial membrane diameter at rest. We neglect the difference in stretch orientation, as in our case the membrane is not stretched unidirectionally but perpendicular to its perimeter. The arc length *s* is calculated according to:

$$s = \int_{-r}^{r} \sqrt{1 + [h'(x)]^2}\, dx \qquad \text{(V)}$$

When deflecting the Actuators membrane in a single step, we calculate a ~10% engineering strain at a deflection height h_{max}= -1.31 mm assuming an ideal 2nd order parabolic shape ($s = 7.15\ \mathrm{mm}, \mathrm{d} = 6.50\ \mathrm{mm}$). Using Hencky's equation (I) and PDMS material data from Dogru et al. (E = 0.87 MPa; v = 0.485) this deflection height is reached at a calculated pressure of about 100 mbar. (Δp = 100 mbar; h_{max}= -1.32 mm; s = 7.15 mm; d = 6.50 mm; e = 10.1%). Our measurement data show a deflection height h_{max}= -1,36 mm at 100 mbar, which is close to the calculated value (Fig. 3b). Moreover, the modelled deflection curve aligns well with the measurement data.

We observed a roughly linear increase in deflection height magnitude with increasing pressure (Fig. 3c). At 200 mbar the membrane contacts the bottom of the culture well. Given the linear behaviour of deflection height and pressure and our pressure controller's resolution of 0.1 mbar, we expect a theoretical resolution of 0.65 µm/ 0.1 mbar for tuning the gap size in our microdevice (assuming a purely linear relationship of deflection height and pressure as shown in Fig. 3c). This resolution depends mostly on the membrane thickness and PDMS material properties. Hence, working pressure and height resolution may vary between Actuator units due to prototyping artefacts. For calculating the engineering strains at different pressures, we fitted the measured data with a 12th order parabola and calculated the arc length s according to (V) (see Fig.S4). We find that the engineering strain increases linearly with pressure (see Fig. 3c). At the working pressure of ~200 mbar, we expect engineering strains of ~23% in the Actuator's membrane.

Supported Lipid Bilayer (SLB) manufacturing protocol

1-palmitoyl-2-oleoyl-glycero-3-phosphocholine (POPC), and 1,2-dioleoyl-sn-glycero-3-[(N-(5-amino-1-carboxypentyl)-iminodiacetic acid) succinyl] (nickel salt) (Ni-NTA-DGS) were purchased from Avanti Polar Lipids, Inc., USA. 98 mol-% POPC and 2 mol-% Ni-NTA-DGS, dissolved in chloroform, were mixed, and dried under nitrogen flow for 20 minutes. The lipid mixture was dissolved in 1 ml 10xPBS (Sigma Aldrich) at room temperature and then sonicated for 10 minutes (USC500TH, VWR, England) to form small unilamellar vesicles (SUVs). The SUV solution was diluted to a final concentration of 125 µM using 10xPBS. The Carrier slide was plasma treated for 10 minutes (PDC-002, Harrick Plasma) and a volume extender was applied over the cell culture well. 70 µl of SUV solution was transferred into the Carrier well and incubated for 20 minutes at room temperature. After incubation, excess vesicles were washed away using 1xPBS (Sigma Aldrich).

Next, SLBs were incubated with Histidine-tagged proteins. B7-1 and ICAM-1 were from Sino Biological (China). I-Ek and MCC peptide were prepared and labelled as described previously[5]. SLBs were incubated for 1 hour and extensively rinsed with PBS afterwards. (Activating conditions (pos): 2 ng I-Ek /MCC, 5 ng B7-1 and 5 ng ICAM-1; resting conditions (neg): 5 ng ICAM-1)

Carrier modules with attached volume extenders were filled to the top with PBS, then 100µL were aspirated and Carriers were stored in the fridge. SLB characterization and cell experiments were performed on the day of SLB generation. Before seeding the cells, PBS was exchanged for HBSS (Sigma Aldrich), then 250 µL was aspirated and the volume extender was carefully removed by hand.

Microdevice reusability

Actuator: Actuator Glass Disc surface and Actuator PDMS Membrane deflection functionality were characterized on new Actuators, Actuators coated with spacer beads (64090-15, Polysciences) and Actuators recycled for reuse according to the protocols described in "Experimental – Spacer bead immobilization" and "Experimental – Actuator recycling". Glass disc surfaces were imaged with a microscope (RVL2-K3, ECHO) in brightfield mode. PDMS membranes were deflected using a pressure regulator (Flow EZ™ Push-Pull, Fluigent) and imaged from the side using a USB digital microscope (see Fig.S10).

Carrier: SLBs were prepared and characterized on new Carriers and on Carriers recycled for reuse (see Fig.S11). Briefly, two different SUV solutions were prepared as described above. The first solution contained only POPC. The second solution was prepared from a mixture of POPC and the tracer lipid 1,2-dioleoyl-sn-glycero-3-phosphoethanolamine labelled with ATTO 488 (DOPE-ATTO488, provided by doc. RNDr. Radek Šachl Ph.D., J. Heyrovsky Institute of Physical Chemistry, Prague, Czech Republic), with a mixing ratio DOPE-ATTO488:POPC of 1:4x10^6. The SUV solutions were diluted with 10xPBS (Sigma Aldrich) to a concentration of 125 µM. To titrate the density of the tracer lipid, POPC+DOPE-ATTO488 SUVs were mixed with POPC SUVs, achieving DOPE-ATTO488:POPC ratios of 1:4x10^6, 1:16x10^6 and 1:32x10^6. Carrier modules were plasma treated for 10 minutes (PDC-002, Harrick Plasma) and 70 µl of SUV solution was transferred into the Carrier well and incubated for 20 minutes in the dark at room temperature. After incubation, excess vesicles were washed away using 1xPBS (Sigma Aldrich). The bilayers were imaged in total internal reflection (TIR) mode on an Axiovert 200 microscope (Zeiss), equipped as described in "Experimental – Supported lipid bilayer imaging" but with a 488nm-diode laser (Oxxius) and a 525/45 BrightLine® single-band pass filter (FF01-525/45-25, Semrock). Bilayers were imaged for 150 frames with a frame rate of 40 Hz and an illumination time t_{ill} = 5 ms. After the first cycle of imaging, the carriers were cleaned as described in "Experimental – Carrier recycling". Subsequently, fresh bilayers were prepared on the cleaned carriers and imaged under the same conditions. To compare the mobility of bilayers before and after cleaning, the SLB microscopy images were analysed similarly as described in "Experimental – Supported lipid bilayer characterization". The ROI was increased to 186px x 188px to include more signals in the analysis and signals were filtered for size (0.75 px < signal size < 1.25 px). For sufficient signal separation, the density threshold was reduced to ~ 0.06 / um² for tracking. The analysis interval was limited to the first 100 frames after the density threshold was met. To account for the faster movement of lipids in comparison to proteins, the tracking radius was increased to 9. Again, only trajectories with a length of at least 5 steps were included in the calculation of diffusion constants.

Our experimental data provided evidence that the Actuator confining surface can be recycled while keeping the actuation mechanism functional (see Fig. S10). We have further shown that our recycling approach allows repeated functionalization of Carrier confining surfaces with mobile SLBs (see Fig. S11). Conclusively, both Actuator and the Carrier module can be recycled which renders our microdevice reusable.

FURA-2 image data processing

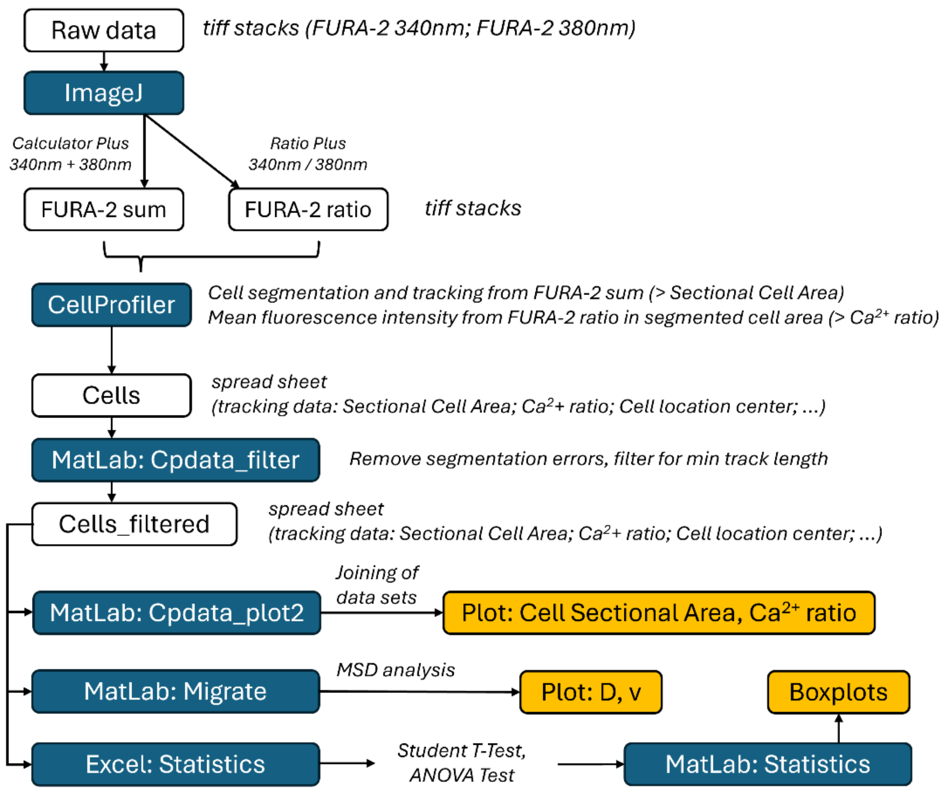

Raw data processing in ImageJ

FURA-2 sum, and FURA-2 ratio stacks are generated from the raw data tiff stacks. We therefore used FIJI (ImageJ 1.53t) with the following plug-ins:

- Ratio Plus: https://imagej.net/ij/plugins/ratio-plus.html
- Calculator Plus: https://imagej.net/ij/plugins/calculator-plus.html

CellProfiler[6] (https://cellprofiler.org/; used version 4.2.1):

FURA-2 sum, and FURA-2 ratio image stacks are processed with a customized pipeline. The pipeline segments cells from the FURA-2 sum images and tracks them over time. The segmented areas, termed Sectional Cell Areas, are saved and used as masks to determine the mean intensity signals in the Ratio images, termed Ca^{2+} ratios. Finally, all data is saved in an excel spreadsheet. This spreadsheet contains information of each identified object in each image frame (ImageNumber, ObjectNumber, AreaShape_Area, Intensity_MeanIntensity_Ratio, Location_Center_X, Location_Center_Y, TrackObjects_Displacement, TrackObjects_DistanceTraveled, TrackObjects_Label).

CPdata_filter

To filter the raw track data, we wrote a MatLab function that allows us to

- filter for tracks continuing to the end of the video (the last frame)
- filter for tracks with a minimal track length
- remove tracks with frequent segmentation errors or incorrect tracking (seen as high standard deviation of segmented cell areas in a single track)
- remove single segmentation errors by an outlier removal method: rmoutliers – "quartiles" from https://de.mathworks.com/help/matlab/ref/rmoutliers.html (removes outliers, not the whole track)

The filtered data are saved in a Spreadsheet again. To check the filtered data the MatLab function plots the cell tracks before and after filtering and saves the filter settings in a Log file.

Example:

Raw data:

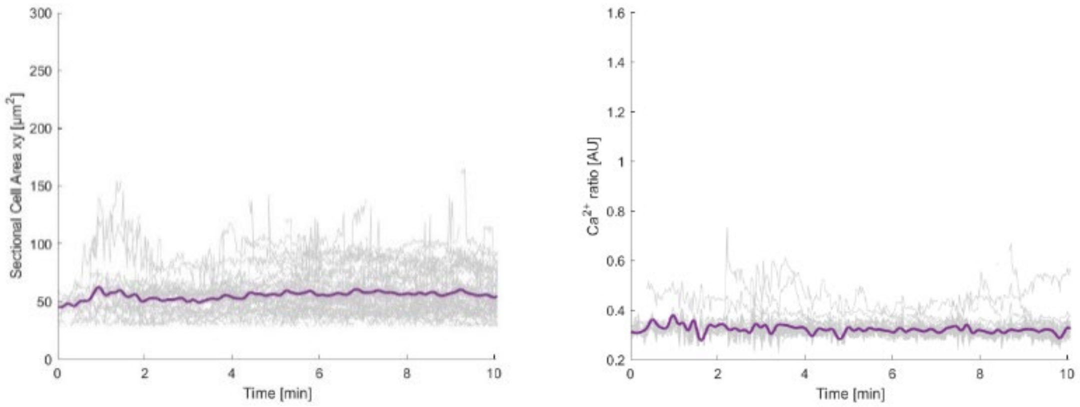

Filtered data:

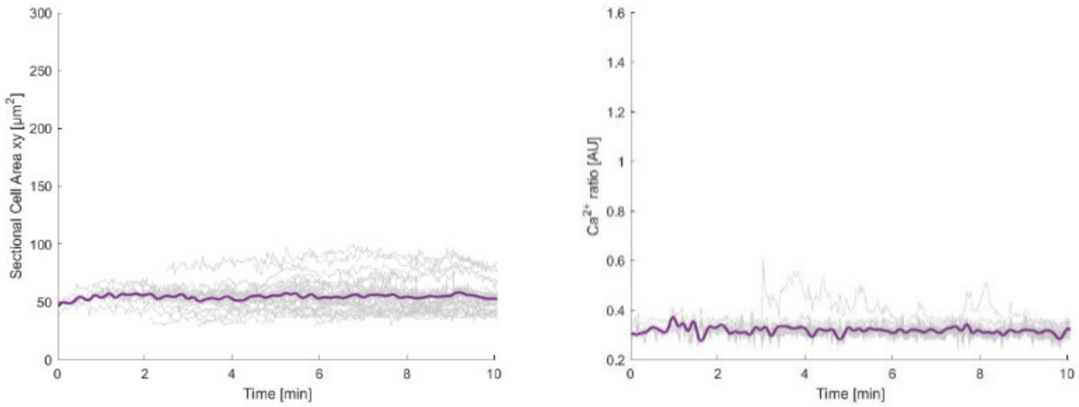

31 from 332 tracks are left after filtering.

Filter Log File:

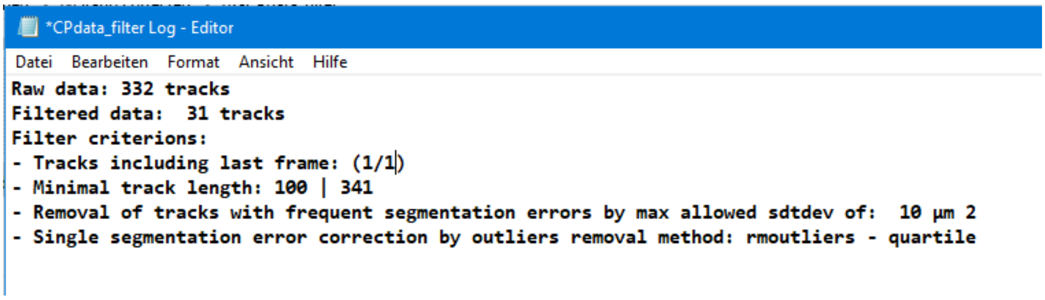


```
Raw data: 332 tracks
Filtered data:  31 tracks
Filter criterions:
- Tracks including last frame: (1/1)
- Minimal track length: 100 | 341
- Removal of tracks with frequent segmentation errors by max allowed sdtdev of:  10 µm 2
- Single segmentation error correction by outliers removal method: rmoutliers - quartile
```

pErk1/2 image data processing

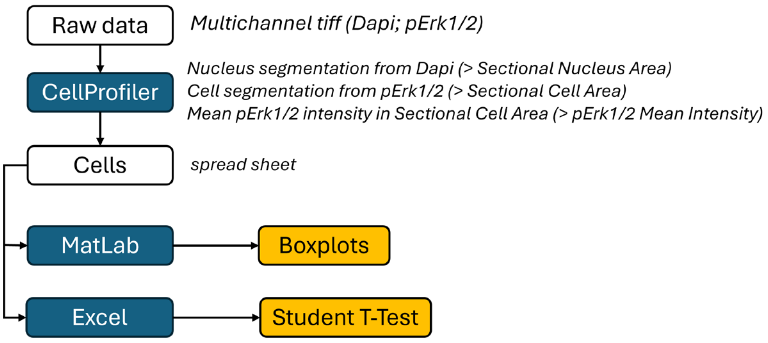

Literature

1 H. Hencky, *ZAMM - J. Appl. Math. Mech. / Zeitschrift für Angew. Math. und Mech.*, 1921, **1**, 81–89.

2 M. S. Zarghamee, *J. Struct. Eng.*, 1990, **116**, 177–187.

3 S. Dogru, B. Aksoy, H. Bayraktar and B. E. Alaca, *Polym. Test.*, 2018, **69**, 375–384.

4 M. Liu, J. Sun, Y. Sun, C. Bock and Q. Chen, *J. Micromechanics Microengineering*, , DOI:10.1088/0960-1317/19/3/035028.

5 J. B. Huppa, M. Axmann, M. A. Mörtelmaier, B. F. Lillemeier, E. W. Newell, M. Brameshuber, L. O. Klein, G. J. Schütz and M. M. Davis, *Nature*, 2010, **463**, 963–967.

6 D. R. Stirling, M. J. Swain-Bowden, A. M. Lucas, A. E. Carpenter, B. A. Cimini and A. Goodman, *BMC Bioinformatics*, 2021, **22**, 1–11.

Supplemental Figures

Fig. S1

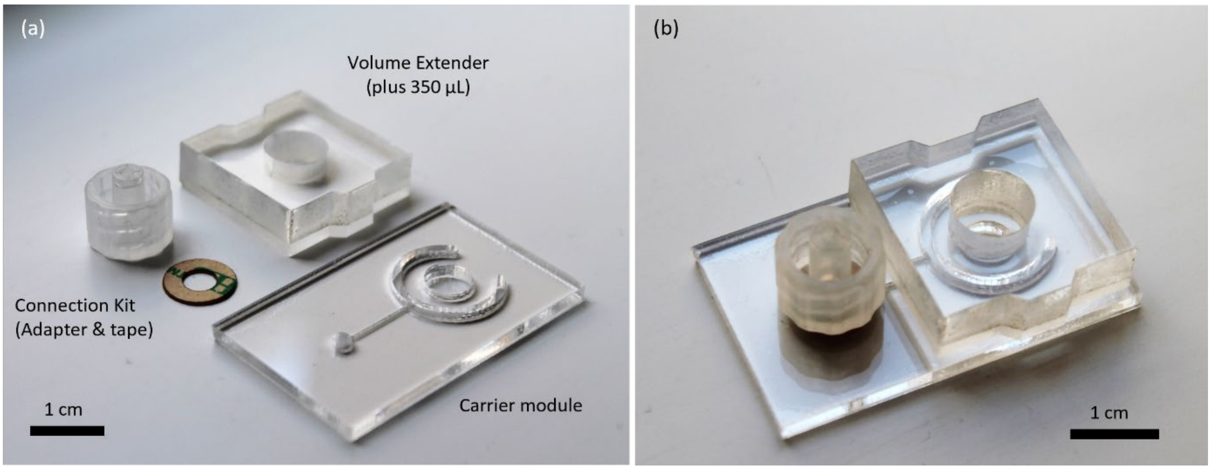

Fig. S1 Volume Extender: (a) individual components before assembly; (b) assembled Carrier module with volume extender and Luer-Lock connector

Fig. S2

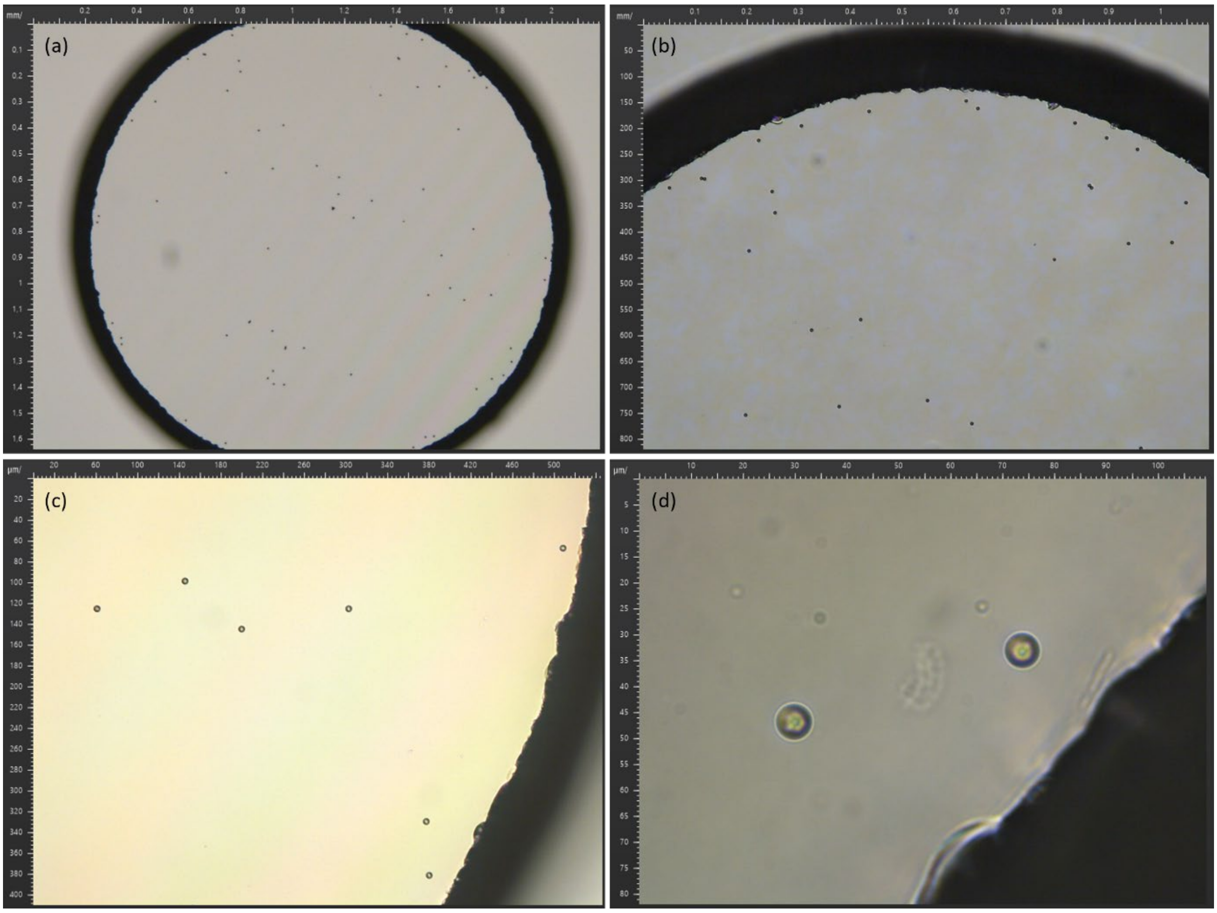

Fig. S2 Spacer beads distribution (6 µm): (a) 5x magnification; (b) 10x magnification; (c) 20x magnification; (d) 100x magnification

Fig. S3

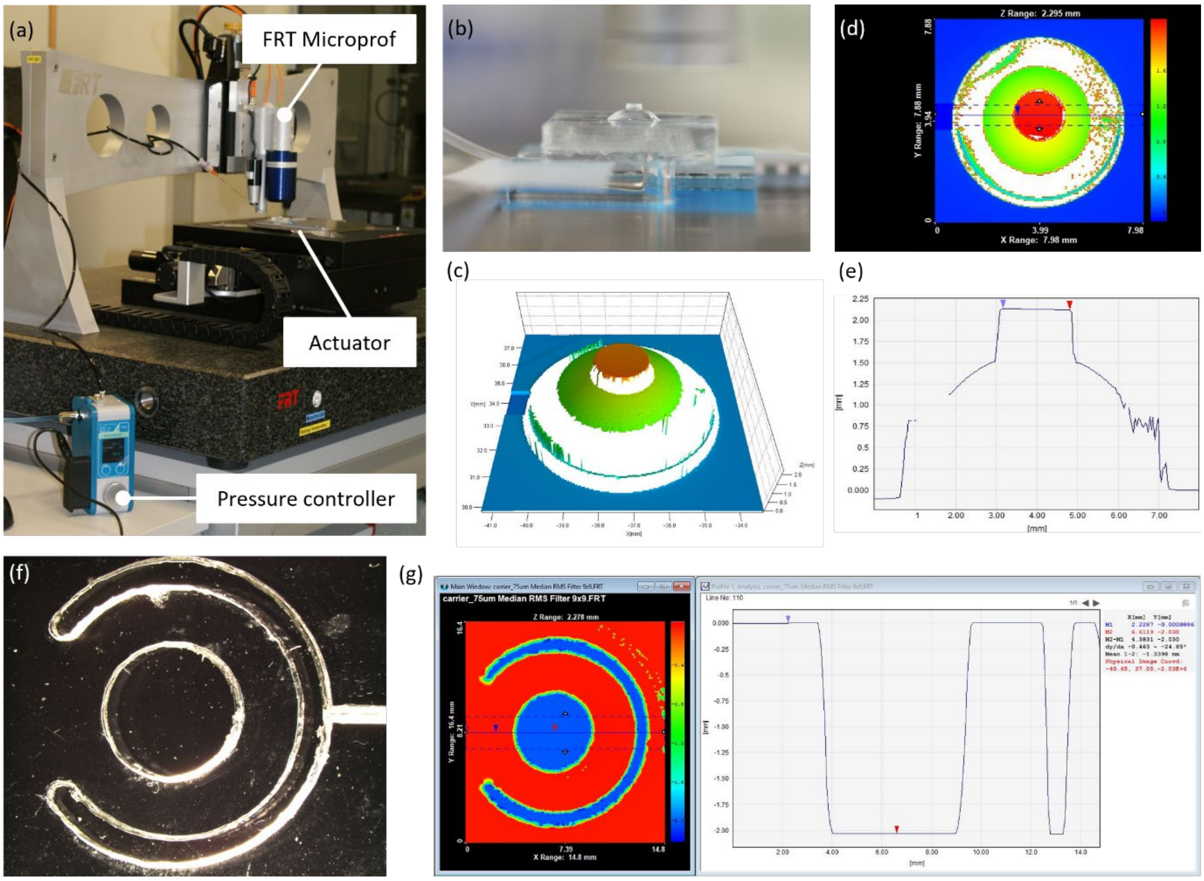

Fig. S3 Membrane topography measurement set-up: (a) Interferometer with loaded Actuator sample; (b) Actuator with deflected membrane up-side down on measurement stage; (c) Isometric view of the measured membrane topography; (d) 2D projection of measured topography data, horizontal line indicates the position taken for the cross-sectional profile data depicted in (e); (f) Carrier surface with round culture well structure and C-shaped vacuum channels; (g) 2D projection of measured topography data (left) and cross-sectional profile (right).

Fig. S4

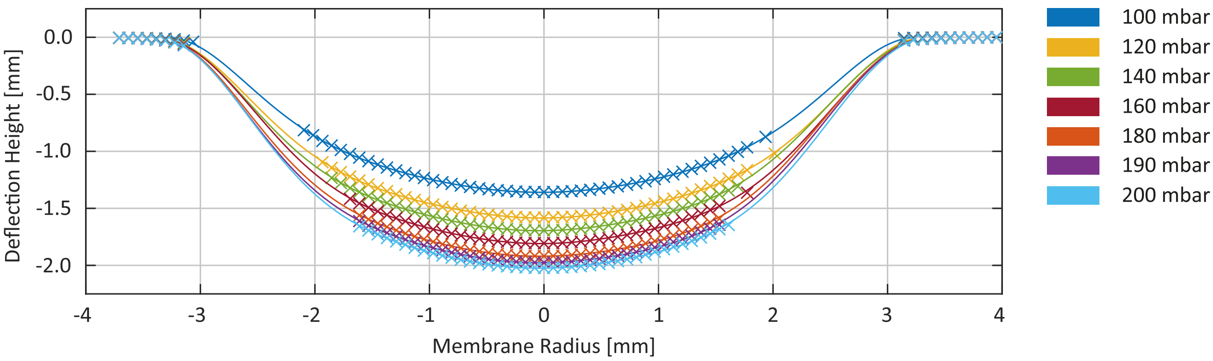

Fig. S4 Membrane Deflection at different working pressures (✕ : measured values; line: fitted 12th order parabola)

Fig. S5

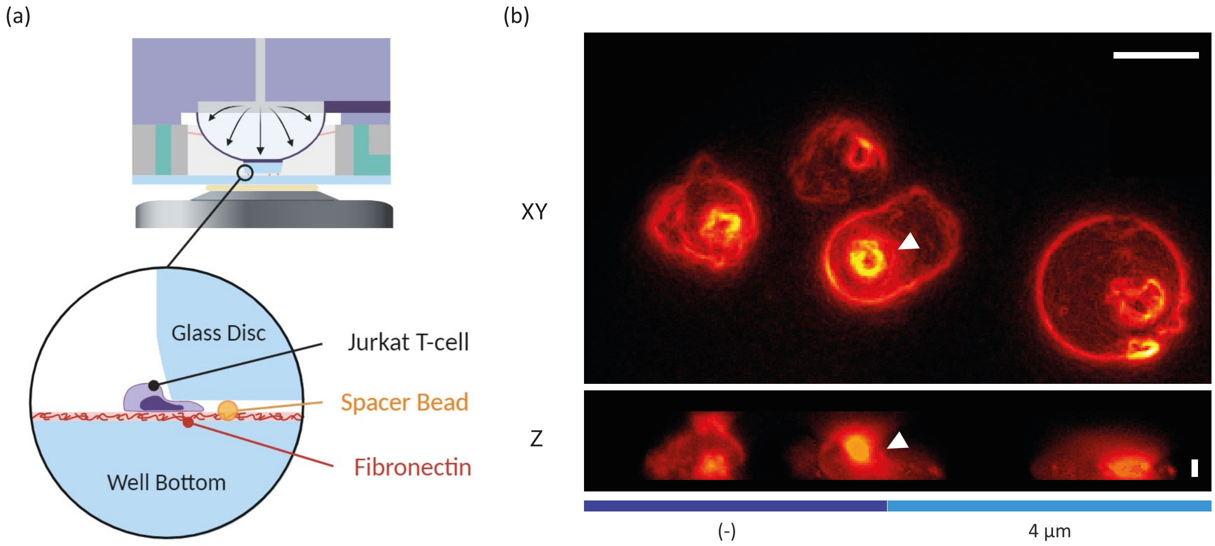

Fig. S5 Confinement precision: (a) Sketch of partial Jurkat T-cell confinement on fibronectin coated surface (b) XY and Z projection of non-confined (-) and confined (4 µm) area at the edge of the Actuator glass disc; the white arrowhead indicates a partially confined Jurkat T-cell (scalebar XY: 10 µm, scalebar Z: 4 µm)

Fig. S6

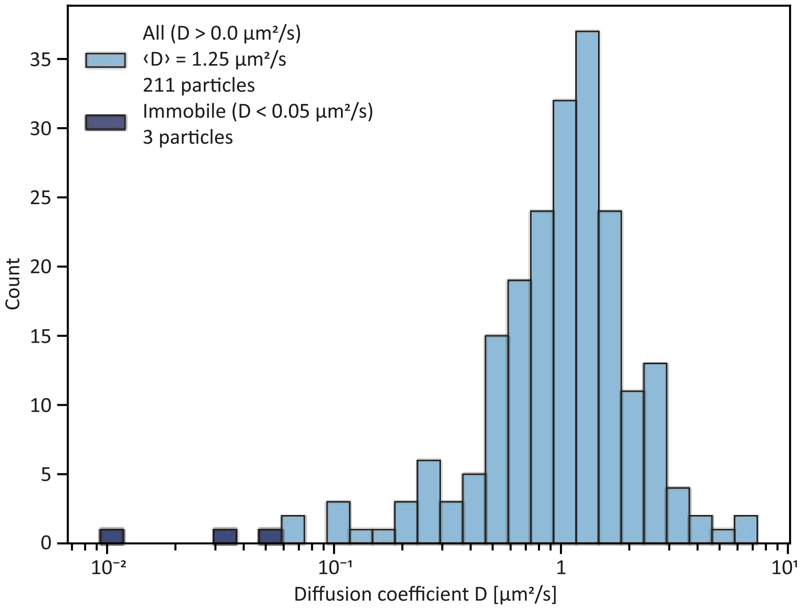

Fig. S6 Diffusion constant distribution of pMHC molecules on the SLB. For each individual pMHC-AF647 track the diffusion constant was determined via the MSD-lag time plot. Molecules diffusing with $D < 0.05$ µm²/s were regarded as immobile. Out of 214 analysed molecules three were found to be immobile.

Fig. S7

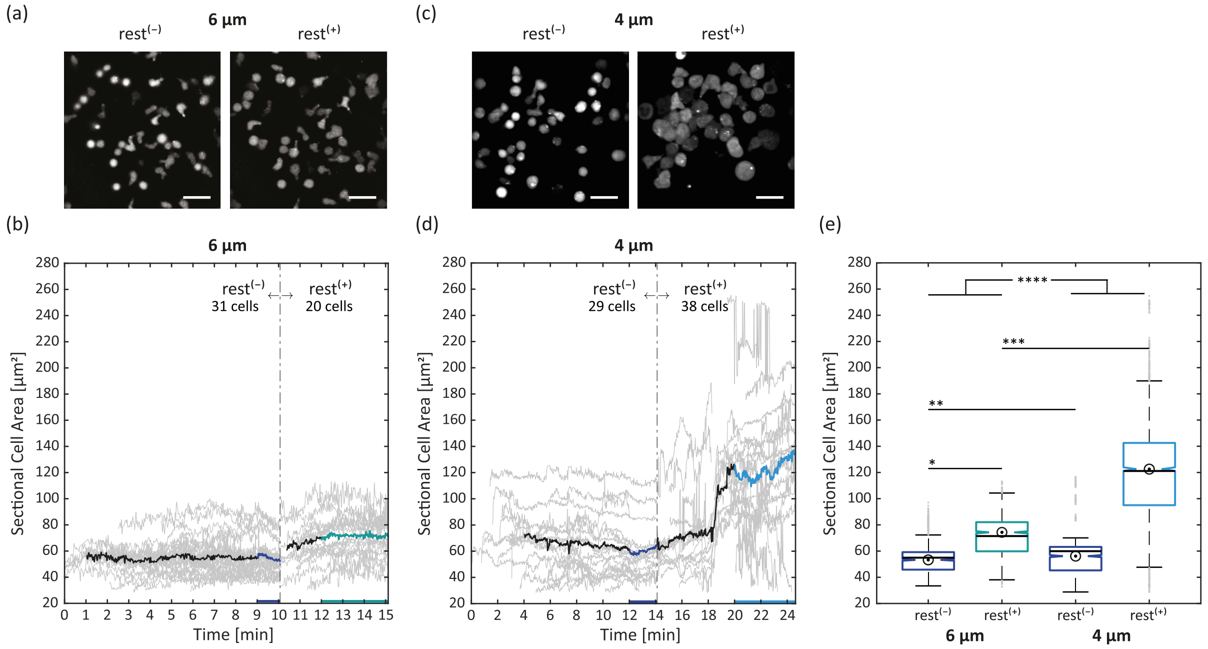

Fig. S7 T-cell confinement to 6 µm and 4 µm: (a) FURA-2 sum image of T-cells on resting SLB, unconfined (rest$^{(-)}$) and confined to 6 µm (rest$^{(+)}$) (scalebar: 25 µm); (b) Sectional Cell Area, before (rest$^{(-)}$) and after confinement to 6 µm (rest$^{(+)}$), for individual cells (grey lines) or averaged over all cells (bold black, dark blue and green line); (c) FURA-2 sum image of T-cells on activating SLB, unconfined (rest$^{(-)}$) and confined to 4 µm (rest$^{(+)}$) (scalebar: 25µm); (e) Sectional Cell Area on activating SLB, before (act$^{(-)}$) and after confinement to 4 µm (act$^{(+)}$), for individual cells (grey lines) or averaged over all cells (bold black, dark blue and light blue); (g) Sectional Cell Area boxplots (bullseye: median; line: mean), pairs of * and *** are significantly different ($p < 0.05$), ** is also significantly different ($p < 0.05$ - note, that 6 µm and 4 µm confinement experiments were conducted on different days and hence, T cells from different mice were used), **** mean difference (rest$^{(-)}$ vs. rest$^{(+)}$) of 6 µm pair is significantly different ($p < 0.05$) compared to 4 µm pair

Fig. S8

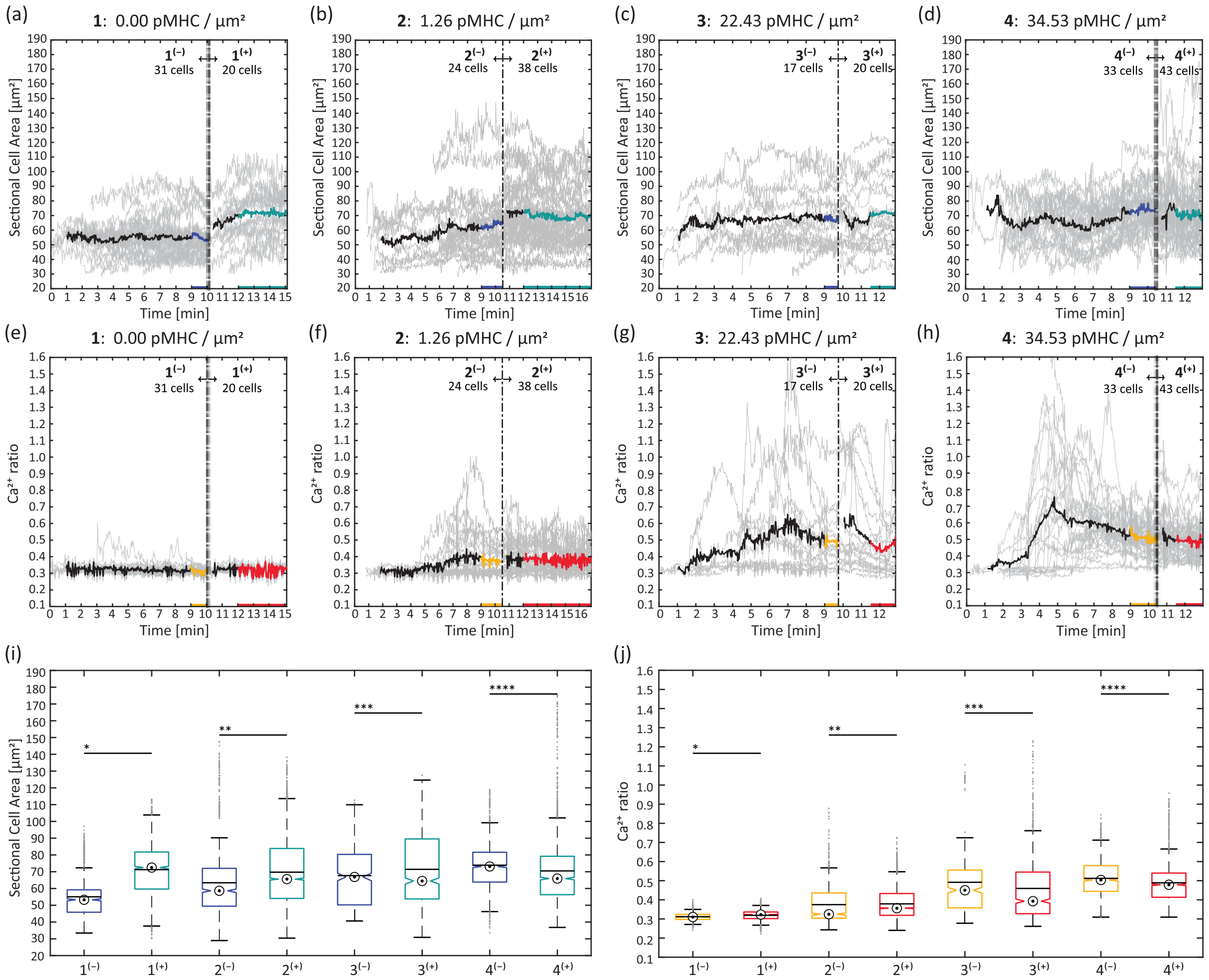

Fig.S8 Effect of bilayer composition on T-cells under 6 µm confinement: (a) - (d) Sectional Cell areas before $^{(-)}$ and after confinement $^{(+)}$, for individual cells (grey lines) or averaged over all cells (bold black, blue and green line); coloured mean tracks indicate data taken for statistical analysis; (e) – (h) Ca^{2+} ratios before $^{(-)}$ and after confinement $^{(+)}$, for individual cells (grey lines) or averaged over all cells (bold black, yellow and red line), coloured mean tracks indicate data taken for statistical analysis; (i) Sectional Cell Area boxplots (bullseye: median; line: mean), Pairwise Student T-Test: All pairs *, **, ***, **** are significantly different ($p < 0.05$), ANOVA Unconfined ($1^{(-)}$, $2^{(-)}$, $3^{(-)}$, $4^{(-)}$): Means are different ($p < 0.05$), Games Howell Unconfined ($1^{(-)}$, $2^{(-)}$, $3^{(-)}$, $4^{(-)}$): All pairwise means are different ($p < 0.05$), ANOVA Confined ($1^{(+)}$, $2^{(+)}$, $3^{(+)}$, $4^{(+)}$): Means are different ($p < 0.05$), Games Howell Confined ($1^{(+)}$, $2^{(+)}$, $3^{(+)}$, $4^{(+)}$): Only pair $1^{(+)}$ - $2^{(+)}$ is different ($p < 0.05$); (j) Ca^{2+} ratio boxplots (bullseye: median; line: mean), Pairwise Student T-Test: Pairs *, ** and **** are significantly different ($p < 0.05$), ANOVA Unconfined ($1^{(-)}$, $2^{(-)}$, $3^{(-)}$, $4^{(-)}$): Means are different ($p < 0.05$), Games Howell Unconfined ($1^{(-)}$, $2^{(-)}$, $3^{(-)}$, $4^{(-)}$): All pairwise means are different ($p < 0.05$), ANOVA Confined ($1^{(+)}$, $2^{(+)}$, $3^{(+)}$, $4^{(+)}$): Means are different ($p < 0.05$), Games Howell Confined ($1^{(+)}$, $2^{(+)}$, $3^{(+)}$, $4^{(+)}$): All pairwise means are different ($p < 0.05$); SLB composition: 1 = rest, 2 = act low (pMHC: 1.26 signals / µm^2), 3 = act mid (pMHC: 22.43 signals / µm^2), 4 = act high (pMHC: 34.53 signals / µm^2)

Fig. S9

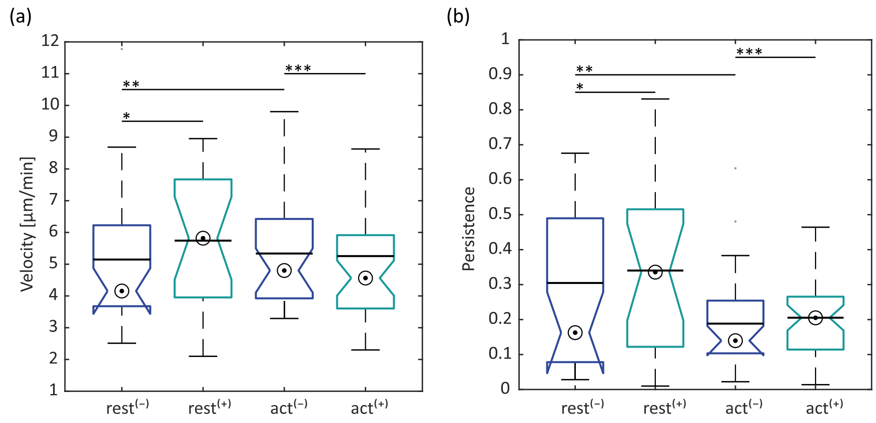

Fig. S9 Migratory behaviour of T-cells under confinement to 6 µm: (a) Average local velocity and (b) directional persistence of T-cells on non-activating (rest) and activating (act) SLB , before (-) and after confinement (+), (bullseye: median; line: mean; N(rest$^{(-)}$) = 31; N(rest$^{(+)}$) = 20; N(act$^{(-)}$) = 33; N(act$^{(+)}$) = 43 cells), */**/*** are not significantly different (p > 0.05) in both subfigures.

Fig. S10

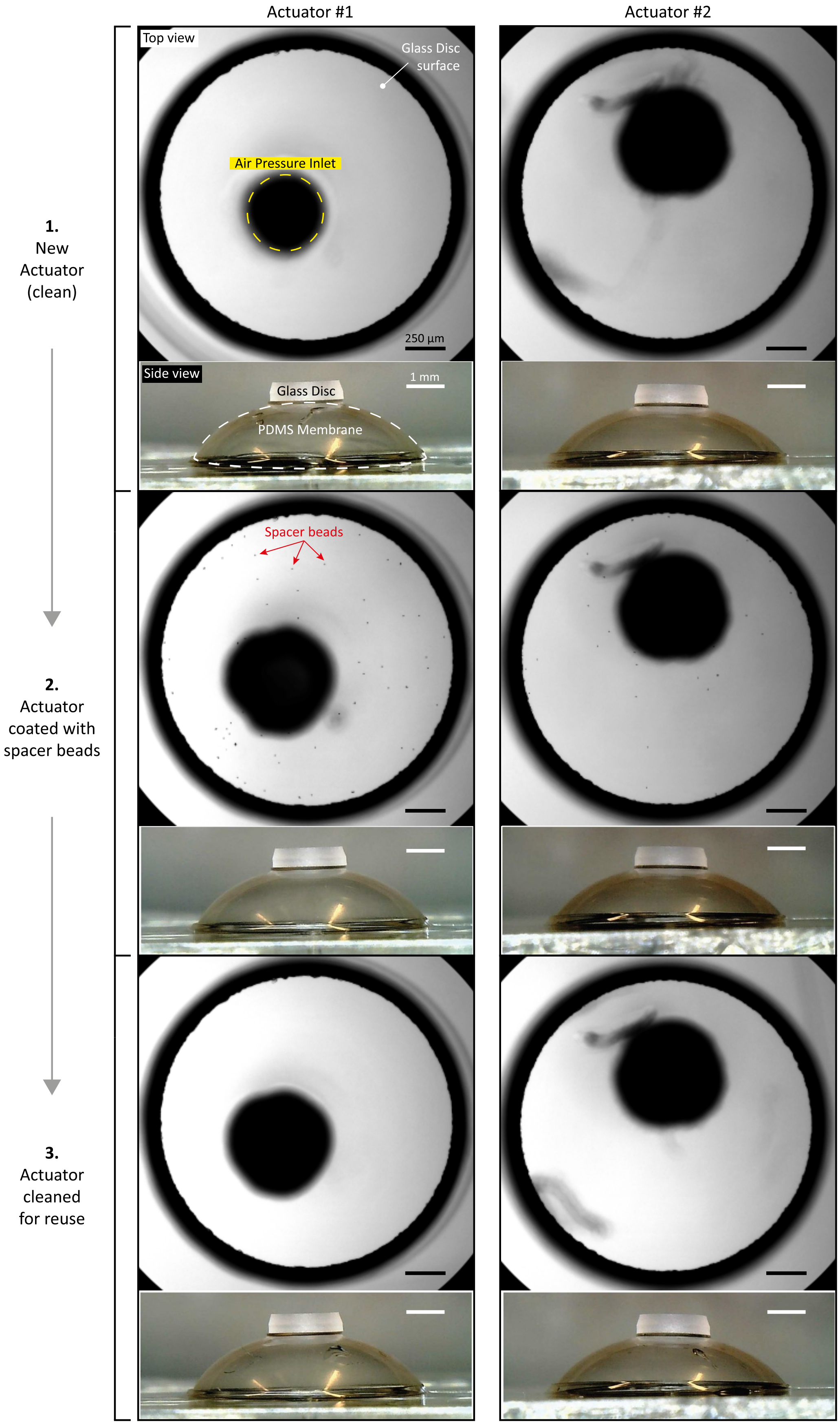

Fig.S10 Actuator reusability: Characterisation of Glass Disc surface and Actuator PDMS Membrane deflection functionality of new Actuators, and the same Actuators coated with spacer beads (d = 6 µm) and recycled for reuse. Confining surfaces can be recycled while keeping the actuation mechanism functional. Each column represents one Actuator module.

Fig. S11

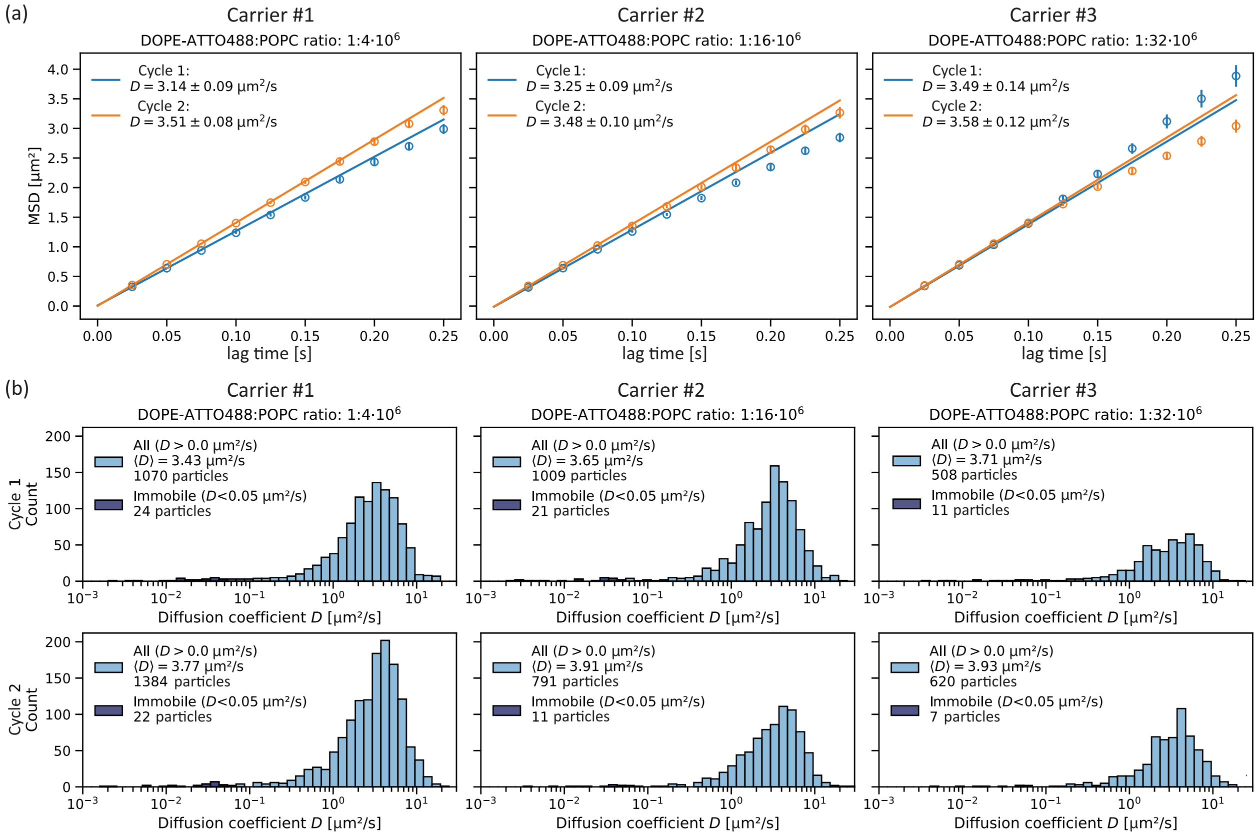

Fig.S11 Carrier reusability: Characterisation of SLBs prepared on new Carriers (Cycle 1) and on the same Carriers after recycling (Cycle 2). (a) Ensemble analysis of Diffusion via MSD-lag time plot of tracked DOPE-ATTO488 shows free Brownian motion of molecules in both cycles. (b) Diffusion constant distribution of DOPE-ATTO488. For each individual DOPE-ATTO488 track the diffusion constant was determined via the MSD-lag time plot. Molecules diffusing with $D < 0.05$ µm²/s were regarded as immobile. Mobile fractions are comparable between cycle 1 and cycle 2. Each column represents one Carrier module.
